# Specialist multidisciplinary input maximises rare disease diagnoses from whole genome sequencing

**DOI:** 10.1038/s41467-022-32908-7

**Published:** 2022-11-07

**Authors:** William L. Macken, Micol Falabella, Caroline McKittrick, Chiara Pizzamiglio, Rebecca Ellmers, Kelly Eggleton, Cathy E. Woodward, Yogen Patel, Robyn Labrum, Genomics England Research Consortium, J. C. Ambrose, P. Arumugam, R. Bevers, M. Bleda, F. Boardman-Pretty, C. R. Boustred, H. Brittain, M. A. Brown, M. J. Caulfield, G. C. Chan, A. Giess, J. N. Griffin, A. Hamblin, S. Henderson, T. J. P. Hubbard, R. Jackson, L. J. Jones, D. Kasperaviciute, M. Kayikci, A. Kousathanas, L. Lahnstein, A. Lakey, S. E. A. Leigh, I. U. S. Leong, F. J. Lopez, F. Maleady-Crowe, M. McEntagart, F. Minneci, J. Mitchell, L. Moutsianas, M. Mueller, N. Murugaesu, A. C. Need, P. O’Donovan, C. A. Odhams, C. Patch, D. Perez-Gil, M. B. Pereira, J. Pullinger, T. Rahim, A. Rendon, T. Rogers, K. Savage, K. Sawant, R. H. Scott, A. Siddiq, A. Sieghart, S. C. Smith, A. Sosinsky, A. Stuckey, M. Tanguy, A. L. Taylor Tavares, E. R. A. Thomas, S. R. Thompson, A. Tucci, M. J. Welland, E. Williams, K. Witkowska, S. M. Wood, M. Zarowiecki, Rahul Phadke, Mary M. Reilly, Catherine DeVile, Anna Sarkozy, Emma Footitt, James Davison, Shamima Rahman, Henry Houlden, Enrico Bugiardini, Rosaline Quinlivan, Michael G. Hanna, Jana Vandrovcova, Robert D. S. Pitceathly

**Affiliations:** 1 Department of Neuromuscular Diseases, UCL Queen Square Institute of Neurology, London, UK; 2 NHS Highly Specialised Service for Rare Mitochondrial Disorders, Queen Square Centre for Neuromuscular Diseases, The National Hospital for Neurology and Neurosurgery, London, UK; 3 Neurogenetics Unit, Rare and Inherited Disease Laboratory, North Thames Genomic Laboratory Hub, London, UK; 4 Dubowitz Neuromuscular Centre, Great Ormond Street Hospital for Children NHS Foundation Trust, London, UK; 5 Department of Neurosciences, Great Ormond Street Hospital for Children NHS Foundation Trust, London, UK; 6 Metabolic Unit, Great Ormond Street Hospital for Children NHS Foundation Trust, London, UK; 7 National Institute for Health and Care Research Great Ormond Street Hospital Biomedical Research Centre, London, UK; 8 Mitochondrial Research Group, UCL Great Ormond Street Institute of Child Health, London, UK; 9 Genomics England, London, UK; 10 William Harvey Research Institute, Queen Mary University of London, London, UK

**Keywords:** Genetic testing, Neuromuscular disease, Metabolic disorders

## Abstract

Diagnostic whole genome sequencing (WGS) is increasingly used in rare diseases. However, standard, semi-automated WGS analysis may overlook diagnoses in complex disorders. Here, we show that specialist multidisciplinary analysis of WGS, following an initial ‘no primary findings’ (NPF) report, improves diagnostic rates and alters management. We undertook WGS in 102 adults with diagnostically challenging primary mitochondrial disease phenotypes. NPF cases were reviewed by a genomic medicine team, thus enabling bespoke informatic approaches, co-ordinated phenotypic validation, and functional work. We enhanced the diagnostic rate from 16.7% to 31.4%, with management implications for all new diagnoses, and detected strong candidate disease-causing variants in a further 3.9% of patients. This approach presents a standardised model of care that supports mainstream clinicians and enhances diagnostic equity for complex disorders, thereby facilitating access to the potential benefits of genomic healthcare. This research was made possible through access to the data and findings generated by the 100,000 Genomes Project: http://www.genomicsengland.co.uk.

## Abstract (web summary)

Whole genome sequencing is emerging as a first-line test for rare genetic diseases. In this study, authors maximise diagnoses by supplementing existing semiautomated analyses with clinically driven reevaluation of genomic data by a specialist multidisciplinary team.

## Introduction

In recent years, the diagnostic bottleneck in rare diseases has moved from accessing genetic testing to data interpretation^1^. Previously, the diagnosis of genetic syndromes was primarily a clinical endeavour based on gestalt, followed by targeted testing of a limited number of genes. Now, physicians rarely select the individual genes to be tested; rather, lists of genes linked to a phenotype (known as ‘virtual gene panels’ [VGPs]) are compiled and applied to whole genome sequencing (WGS) or exome data to filter the large number of variants within an individual’s genome. In England, The 100,000 Genomes Project (100kGP) has combined clinical WGS with availability of data for research, and has formed the basis for the establishment of a National Health Service Genomic Medicine Service (NHS GMS)^2,3^. The NHS GMS enables individuals with rare diseases to access WGS as part of routine diagnostic care and to share their data with researchers in a secure genomic ‘library’. Through the NHS GMS, England will sequence and process a further 500,000 genomes via a central facility. This places England in a relatively unique situation; the host of a centralised model that couples large scale datasets and high-throughput bioinformatics capabilities, with local clinical analysis and interpretation. In contrast, other healthcare systems utilise more integrated localised services, while some adopt a private healthcare model, whereby laboratory providers undertake sequencing and interpretation remote to the clinical facility. Regardless of the healthcare system, balancing widespread introduction of WGS with sufficient analysis of these large datasets is a significant challenge. Genomic medicine itself can be considered an emerging medical discipline that uses genomic data to inform clinical management^4^. In this regard, it is similar to the use of medical imaging by radiology. In rare diseases, genomic medicine roles may be fulfilled by clinical geneticists whose generalist training confers knowledge across a wide-range of presentations, but may also include subspecialists, e.g. cardiologists with genomic training in an inherited cardiac disease service. In the NHS GMS and similar models, ‘mainstream’ medical specialists, whose primary training is not in genomic medicine, are increasingly the physicians tasked with ordering WGS and acting on results. Though mainstream clinicians may accept WGS reports as definitive results, the reality is more complex for the following reasons: (1) VGPs may filter out diagnostic variants if a sufficiently broad approach is not adopted—indeed work from the 100kGP pilot suggests VGPs overlook up to 40% of possible diagnoses when additional bioinformatic strategies are not applied^5^; (2) a report may refer to a variant of uncertain significance (VUS), which cannot be reported as an ‘actionable’ finding due to inadequate supporting evidence, but may represent the correct diagnosis in some cases; (3) post-WGS investigations may meaningfully improve the diagnostic yield, e.g. ‘reverse phenotyping’ (the reassessment of the phenotype and additional clinical investigations to validate whether a variant is relevant to the patient), functional validation (by a translational or research scientist), and cutting-edge bioinformatic approaches may generate additional diagnoses for patients^6^. These complexities require input from a specialist multidisciplinary team (MDT); offering a standardised approach when ‘no primary findings’ (NPF) are reported, supporting mainstream physicians, and maximising the utility of diagnostic WGS for patients with rare and complex diseases.

Researcher identified potential diagnoses (RIPDs) in WGS data improve diagnostic rates and are essential in establishing novel genetic conditions^7,8^. However, owing to discrepancies in research interests and funding, lack of access to detailed clinical data, and variable patient involvement in research, we believe RIDPs cannot offer a systematic or equitable solution to the challenge of unsolved WGS. Separately, given their burgeoning workload, burdening clinical scientists with additional analysis following initial interpretation is untenable. Consequently, we believe staffing dedicated specialist MDTs to review complex cases, and undertake confirmatory functional studies when required, should be considered.

Primary mitochondrial diseases (PMDs) represent a group of inherited disorders that arise from mutations in mitochondrial or nuclear DNA (mtDNA or nDNA), leading to defects in oxidative phosphorylation or other aspects of mitochondrial functioning. PMDs are a par exemplar for the new genomic medicine paradigm. They manifest with a broad range of clinical phenotypes, which renders targeted genetic testing impractical, and supports an inclusive approach to the differential diagnosis^9^. Unlike diseases with a stand-out presenting feature or ‘diagnostic handle’, PMDs are more representative of the broad diagnostic categories encountered in medical and paediatric clinics, which can exhibit significant variability and overlap in their genetic bases. PMDs crosscut medical specialities and highlight the benefits of having a genomic medicine specialist/clinical geneticist in rare disease MDTs, ensuring a comprehensive overview of potential causes for the presentation is maintained.

In this work, we propose an integrated clinical solution for patients with NPF following WGS analysis within English national healthcare genetic services. Through personalised re-analyses, led by a genomic medicine clinician and bioinformatician, we improve the genetic confirmation rate and enable mainstream clinicians and patients to maximise the diagnostic utility of their data.

## Results

One hundred and two adult patients from 96 families underwent WGS (55.9% [57/102] female, 44.1% [45/102] male), following negative routine investigations for PMD (testing for recurrent variants in mtDNA and commonly affected nDNA genes—see Supplementary Table 1). The age range was 17y–81y with a mean age of 47.3y. Modified Nijmegen scores (Supplementary Table 2), initially developed to assess the likelihood of an underlying diagnosis of PMD in young patients, were assigned to all cases; 26.5% (27/102) were classified as having ‘definite mitochondrial disease’, 49% (50/102) had ‘probable mitochondrial disease’, and 24.5% (25/102) had ‘possible mitochondrial disease’. The mean score was 6.2 and mode was 6 (both probable mitochondrial disease scores). The most common family structure recruited was singleton 44.1% (45/102), followed by trio 27.4% (28/102), duo 21.6% (22/102), and larger structures of 4–6 individuals 6.9% (7/102).

**Fig. 1:**
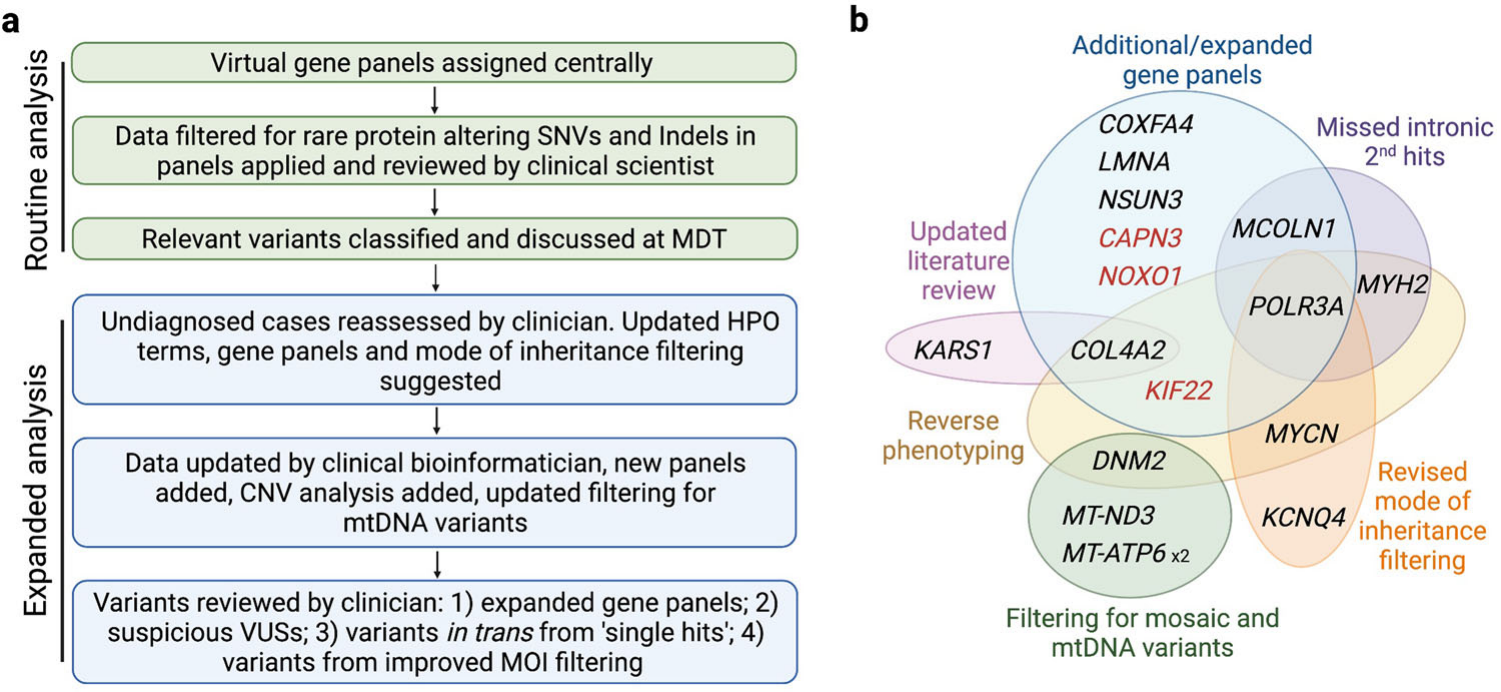
Methodology for data analysis and themes identified in additional diagnoses. **a** Methodology adopted in study. Green panels = routine analysis in 100,000 Genomes Project clinical arm with interpretation undertaken by clinical scientist; Blue panels = enhanced clinician and bioinformatician involvement. **b** Venn diagram representing the factors contributing to new findings (red genes are strong VUSs). MOI, mode of inheritance; SNV, single nucleotide variant.

Routine analysis (Fig. 1a) achieved a molecular diagnosis in 16.7% (17/102) of individuals (see Patients 1–17 in Supplementary Table 3). This diagnostic rate was relatively low when compared with previous studies, emphasising the complexity of this well investigated cohort and raising the suspicion of diagnoses overlooked by a routine semi-automated method^10^. Of these, 6/17 (35.3%) were confirmed to have a PMD, 5/17 (29.4%) had a mutation in a non-mitochondrial neurogenetic or neurodevelopmental gene, 3/17 (17.6%) had a muscular dystrophy or myopathy, 2/17 (11.8%) had a non-mitochondrial cardiomyopathy gene mutation, and 1/17 (5.9%) had a non-mitochondrial deafness gene mutation. Three out of 17 (17.6%) had a genetic diagnosis that only partially explained the phenotype (only explaining deafness, cardiomyopathy, and epilepsy/intermittent weakness respectively in a more complex phenotype). This suggests the possibility of a dual genetic diagnosis, so called ‘double trouble’, resulting in a compound phenocopy of PMD, although a second genetic variant was not identified.

Following initial analysis, customised re-analysis was undertaken following a comprehensive review of all phenotypes and pedigrees. Based on this re-evaluation, data were updated after the application of supplementary, VGP-based filters (Fig. 1a). These included: (1) disease-relevant genes with insufficient evidence to meet the threshold for diagnostic testing (known as ‘Amber’ and ‘Red’ genes in the 100kGP); (2) VGPs missed in the initial assessment (that following phenotype review were felt to warrant inclusion); (3) reassessment of VUSs, identified during the initial analysis; (4) assessment for in trans variants, where a strong heterozygous candidate was identified in a recessive gene; (5) revised mode of inheritance pattern following re-review of family pedigrees. The data were interrogated for copy number variants (CNVs) and mtDNA was examined using customised analyses (see Methods). This resulted in a further diagnostic uplift of 14.7% (15/102) and an additional 3.9% (4/102) candidate diagnoses (highly suspicious VUSs in known or newly established genes), see Supplementary Table 3, Patients A to S. To assess whether better initial phenotyping could have delivered diagnoses in a more automated manner, improved Human Phenotype Ontology (HPO) terms and family structures were employed for Exomiser re-analysis and results were compared with the Exomiser data derived from the initial HPO terms submitted. Exomiser re-analysis did not result in improved prioritisation of variants detected in customised analysis, suggesting that it may not be realistic to automate identification of more complex diagnoses at present. Importantly, work from the 100kGP suggests that 88% of WGS diagnoses are present in the top five Exomiser ranked variants^5^; however, although the majority of routine diagnoses in our cohort would have been captured by Exomiser, only 2/19 (10.5%) of additional cases (*COXFA4*— rank 1, and *KCNQ4*—rank 4) would have been solved. For the two dual diagnosis cases, only one variant was prioritised to top five by Exomiser for each (*KARS1* and *CAPN3)*. All variants were validated using Sanger sequencing and variant classification was verified by a clinical scientist. No causative CNVs were identified. A detailed discussion of each case is included in Supplementary Notes in accompanying Supplementary Information.

**Fig. 2:**
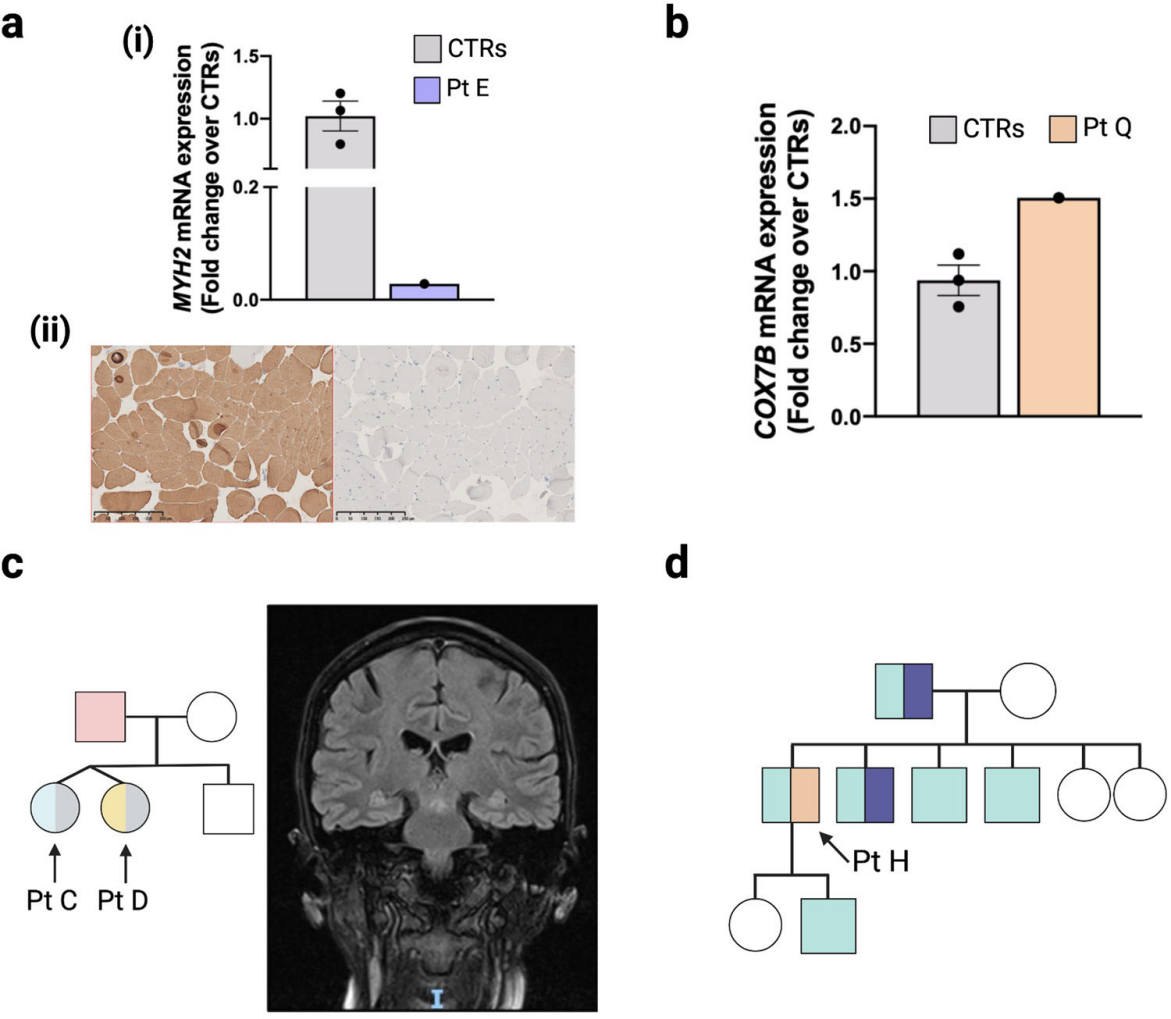
Factors contributing to additional diagnoses, part 1. **a** A novel non-coding *MYH2* variant, c.4188-23T>A, with elevated splicing prediction scores was detected in trans with a loss-of-function variant (c.30del). (i) *MYH2* transcripts were reduced (>99%) in the muscle tissue of Patient (Pt) E compared with controls (CTRs). This loss of *MYH2* was supported by reverse phenotyping undertaken by pathology; *MYH2* is expressed in 2A fast fibres. (ii) Left image—immunostaining for myosin heavy chains showed marked slow fibre predominance. Right image—labelling for 2A fibre specific antibody ‘7.5.2B’ was negative suggesting complete loss of 2A fibres. Each staining was performed in two serial sections. **b** Increased expression of *COX7B* transcripts in Pt Q (c.40 + 5G>A) fibroblasts; we consider this a suspicious VUS—see Supplementary Data for further functional work. **c** Pedigree for Pt C and D (Pink = cardiomyopathy, Grey = myopathy, Blue = spastic gait, Yellow = sensory neuropathy). These twin females initially presented with myopathy and a paternal history of cardiomyopathy, raising the possibility of dominant disease. Pt C had normal genetic testing for congenital myopathy and myasthenic syndromes but had abnormal respiratory chain enzyme activities suggestive of mitochondrial dysfunction (reduced complex I, II, III and IV activity). Pt C developed a spastic paraparesis and white matter disease in later adulthood, while Pt D developed a sensory neuropathy. However, the siblings’ phenotypes have become more similar over time, so were re-evaluated as a recessive neuropathy leading to the diagnosis of compound heterozygous *POLR3A-*related disease (including a heterozygous intronic mutation). MRI brain from Pt C (right panel) demonstrated symmetric signal increase within the mid brain, superior cerebellar peduncles, and dentate nuclei (highly suggestive of a *POLR3A* disorder). **d** Reinterpretation of Pt H’s pedigree (Blue = deafness, Purple = cardiomyopathy, Orange = axonal sensory polyneuropathy) suggested there are multiple disorders in this family, and a novel variant in *KCNQ4* p.(Tyr101_His102insLeuValTyr) was confirmed to segregate with the deafness phenotype. This underlines the importance of considering ‘double trouble’ especially for common genetic diseases, e.g. non-syndromic hearing loss.

**Fig. 3:**
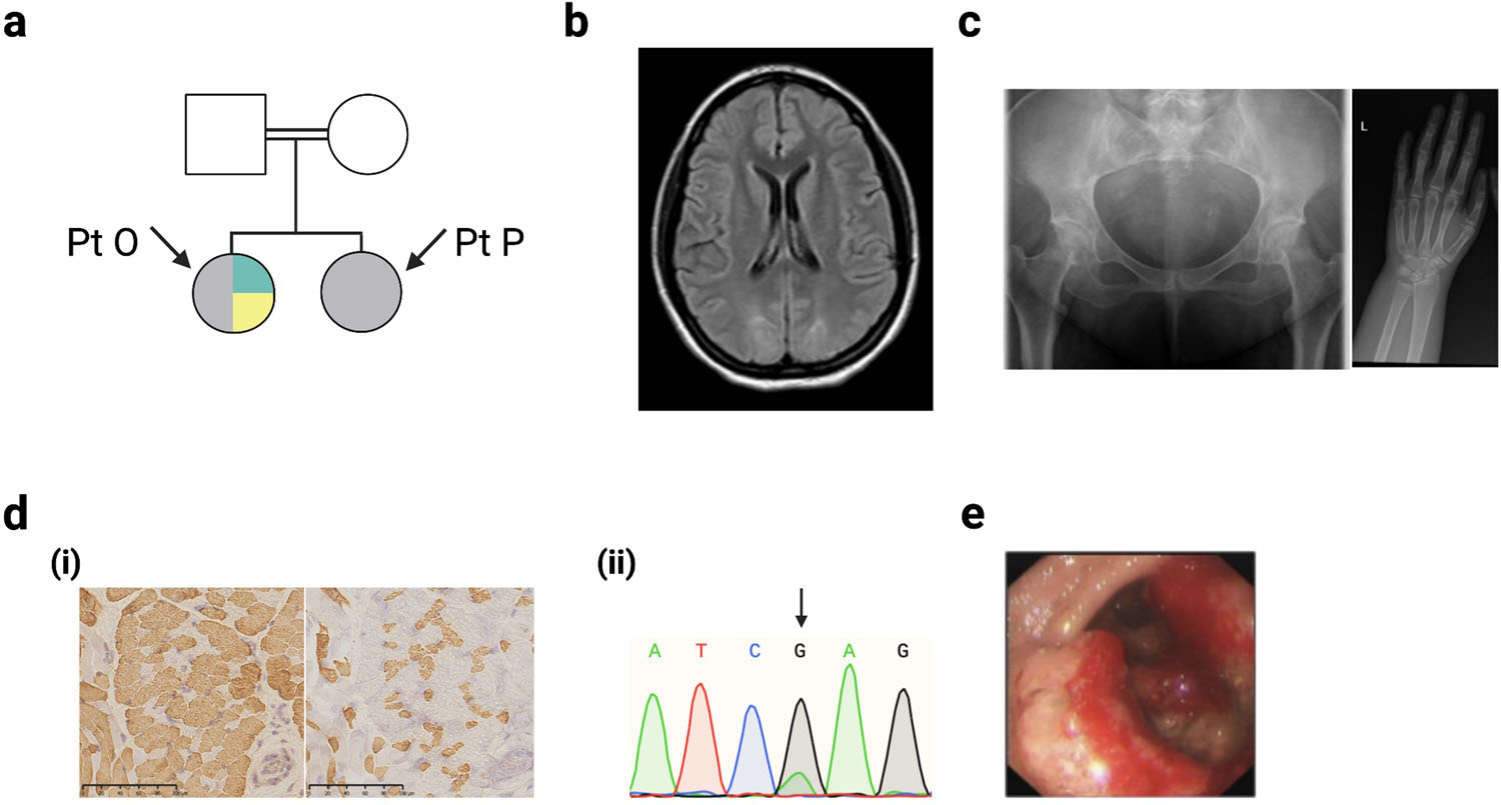
Factors contributing to additional diagnoses, part 2. **a** Reinterpretation of this pedigree suggested multiple conditions present in Patient (Pt) O (Grey = reversible COX deficiency, Teal = dysmorphism and intellectual disability, Yellow = myalgia and proximal weakness). Both siblings have a homozygous variant in *CAPN3,* which can cause a late-onset muscular dystrophy, whereas only Pt O had a de novo variant in *MYCN* that explained her dysmorphism, microcephaly, cardiac disease, and developmental delay. We suspect a cryptic third mutation may exist in this family to account for the COX deficiency. **b** MRI brain in Pt G demonstrated occipitoparietal white matter changes in keeping with the recently identified phenotypic spectrum of *COL4A2*, now known to cause seizures and exhibit variable penetrance. **c** A *KIF22* variant was identified in Pt R whose phenotype included midface flattening, velvety skin, and unusual hands. Skeletal survey suggested a mild version of spondyloepimetaphysial dysplasia with joint laxity (right radiograph demonstrates elongated femoral necks, left radiograph shows long and tapered fingers) and review of the history revealed recurrent joint subluxations. **d** Improved data filtering enabled identification of a de novo mosaic mutation in the myopathy/mtDNA maintenance gene *DNM2*. (i) Muscle biopsy supported this diagnosis, showing fibre size disproportion with mild overall fast fibre predominance (left = fast fibre staining, right = slow fibre staining). Each staining was performed in two serial sections. (ii) Black arrow highlights the mosaic nucleotide in the Sanger sequencing read out. **e** *MCOLN1* variants (one coding, one intronic) were identified in two affected non-dysmorphic siblings. As shown, the male sibling developed an unusual, large ulcerated gastric tumour. Given this disorder leads to achlorhydria, elevated gastrin, and implicates the same protein targeted by *H. pylori’s* virulence factor, we postulate it increases risk of gastric neoplasia.

Distinct themes emerged from our findings (Fig. 1b). First, in 5/15 patients (three families) an intronic second hit was missed in a recessive gene (*MCOLN1, POLR3A, MYH2*), despite some of these variants previously being reported in the literature. This highlights the challenge of interpreting intronic variants without RNA data, even with improving splicing prediction tools^11,12^. Indeed, of the intronic variants identified only *MYH2* had a highly elevated Splice-AI score (delta score acceptor gain 0.99). Identification of these intronic variants was therefore primarily driven by the recognition of a strong correlation between the gene in which a heterozygous variant was identified and the clinical presentation documented by the clinician. Second, functional validation may be required for specific variants. Of the non-coding variants identified one (*MYH2*) was absent from the literature. A translational scientist undertook functional validation (qPCR) which demonstrated a > 99% reduction in *MYH2* transcript levels versus controls [(Fig. 2a, panel (i)], see Supplementary Notes for further details. We also undertook functional investigation of a non-coding heterozygous VUS in *COX7B*, which showed significantly upregulated transcript levels (Fig. 2b), and a collaborator assisted in tRNA methylation analysis for a variant in *NSUN3* (see Supplementary Notes). Third, applying additional gene panels following further evaluation of the clinical phenotype (*POLR3A, COL4A2, KIF22, CAPN3*), and inclusion of genes previously considered to have insufficient evidence to meet the threshold for diagnostic testing (*COXFA4, NOXO1, NSUN3*), contributed towards seven new findings. This supports the utility of VGPs in diagnostic WGS, but underlines that all VGPs with a strong link to the phenotype must be employed, and VGP content must be updated regularly. We do not support sequentially adding VGPs with increasingly tenuous relations to the underlying phenotype. To do so may inadvertently lead to problematic incidental diagnoses. Rather, the complete phenotype and gene content should be carefully considered when selecting VGPs to apply, thereby ensuring the most appropriate analysis is undertaken. Fourth, interrogation of the family pedigree and variant segregation prompted a diagnosis in three cases: (1) Fig. 2c (Patients C/D); (2) Fig. 2d (Patient H); (3) Fig. 3a (Patients O/P). Fifth, reverse phenotyping contributed to five cases: brain MRI review was in keeping with the CNS phenotype exhibited by Patient G (Fig. 3b) and Patient C (Fig. 2c); skeletal survey and updated clinical history suggested a mild variant of Hall-type spondyloepimetaphysial dysplasia with joint laxity (Fig. 3c, Patient R); muscle biopsy supported the pathogenicity of a mosaic variant in *DNM2* (Fig. 3d panel (i), Patient I) and compound heterozygous variants in *MYH2* in Patient E [Fig. 2a panel (ii)]; and Patient O dysmorphology was in keeping with a mild variant of Feingold syndrome type 1. Sixth, improved filtering strategies identified three heteroplasmic mtDNA variants and one mosaic variant in *DNM2* [Fig. 3d panel (ii)]. Seventh, updated information from the literature contributed to two findings: (1) *KARS1* (Patient M)—reporting of a broader phenotype and publication of variants in other individuals improved interpretation; (2) *COL4A2* (Patient G) was previously associated with porencephaly, but has recently been established to cause milder disease and exhibit variable penetrance^13–15^. In two cases, a dual diagnosis with variants in two genes was established (Patients M and O).

The mean time from presentation to diagnosis was 21.3 years in patients diagnosed via the routine approach, and 30.9 years for those diagnosed via the expanded approach. In comparison, the mean diagnostic odyssey in the 100kGP pilot was 6.25 years, underlining the complexity of our cohort^5^. Importantly, there were direct management implications for the 15 newly confirmed diagnoses: one was eligible for a clinical trial for an antisense oligonucleotide; one was eligible for a small molecule drug trial; five had screening for systemic complications stepped down; six families had affected individuals of childbearing age with new reproductive options; and multisystem screening was necessary in five cases. We suggested malignancy screening in one family [Patient (Pt) A and B, Fig. 3e] who has mutations in *MCOLN1* and a history of gastric neoplasia (see Supplementary Notes).

## Discussion

Our clinically-directed approach to WGS facilitated an improved diagnostic rate from 16.7 to 31.4% in patients with suspected PMDs, with a potential further increase to 35.3% when suspicious VUSs are included. While there has been a shift towards automating data interpretation, such approaches are insufficiently sensitive to diagnose more challenging cases at present. Our research underlines the importance of embedding genomic medicine clinicians within the infrastructure of diagnostic genetic services. Previous studies involving clinical geneticists in the diagnostic process have achieved strong diagnostic rates^16,17^, but owing to staffing constraints it is unlikely that every patient undergoing WGS will be reviewed by a geneticist. This leads us to advocate for a specialist MDT approach, in which high-throughput clinical scientist analysis is supplemented by additional clinical and bioinformatic oversight for undiagnosed cases. The advantage of such a service is further exemplified when comparing our work to a related study of WGS in PMDs, which relied on a researcher-led approach^18^. Of the 10 newly diagnosed patients overlapping with our study, seven were overlooked by the researcher-led strategy (see Supplementary Table 5). This emphasises the value of a specialist MDT with both in-depth clinical knowledge and the capacity to provide bioinformatic optimisation to identify diagnoses missed by routine and research-led approaches.

**Fig. 4:**
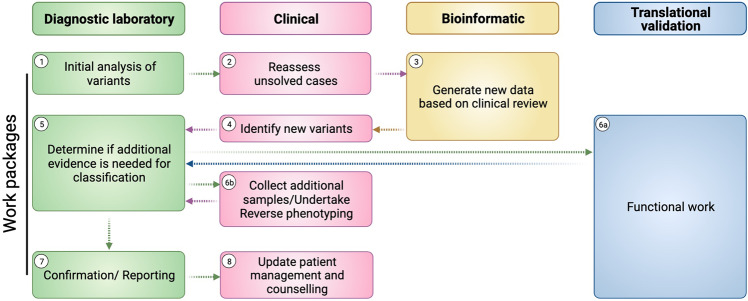
Model for a specialist genomic MDT. We suggest that an evolved specialist genomic MDT model is needed for complex cases. After initial analysis of variants (1), a genomic medicine clinician should re-evaluate the case (2), and data should be updated to address the nuances of the patient presentation (3). The clinician can then review new variants (4), and feed promising variants back to the diagnostic laboratory (5) who, where necessary, would work with a translational scientist and clinician (6a and 6b) to ensure maximum evidence is gathered to confirm the pathogenicity of variants (7), ultimately resulting in improved patient management and counselling (8).

We suggest that evolving the specialist genomic MDT (clinician, bioinformatician, and translational scientist roles) will provide a robust standard of care that improves clinical gains, supports mainstream clinicians, and enhances collaboration with research teams without overwhelming diagnostic laboratories (Fig. 4). The importance of the clinical-research interface is emphasised by data suggesting that an extra 10% of diagnoses could have been secured during the 100kGP pilot if functional work by a translational or research scientist was available^5^. We suggest specialist MDTs form the basis of a hub-and-spoke model, with a nodal team serving multiple rare disease services. Of rare disease patients, those with complex, multisystem, and overlapping phenotypes are most likely to benefit from this approach (e.g. neurodevelopmental disorders, metabolic, and neurological/neuromuscular syndromes). Given the volume of WGS emerging, up scaling this approach will be demanding and focusing on the patients most likely to see a diagnostic uplift is justified. We acknowledge the potential challenge of staffing specialist MDTs and that the proposed model is not readily applicable to all healthcare systems (e.g. the private sector). However, we would argue that our core findings (i.e. the value of establishing specialist genomic MDTs to scrutinise data for diagnoses in complex patients) is relevant to all healthcare environments. It is certainly possible to provide diagnostic WGS at scale without this approach; however, in its absence we compromise the quality of analysis and clinical potential the data holds. The success of our method in identifying missed diagnoses is underpinned by the availability of in-depth, patient-specific knowledge and reverse phenotyping, coupled with genetics and bioinformatics expertise in PMDs. NHS England commissions rare disease care into Highly Specialised Services (HSSs), which provide clinical and diagnostic expertise for large populations of patients. These HSSs combine a breadth and depth of knowledge with deeply phenotyped patient cohorts, in addition to their WGS datasets. As our data demonstrates, coupling such expertise with direct patient access is crucial to realise the potential benefits of diagnostic WGS. Importantly, telemedicine and digital platforms can extend outreach to ensure equitable access to such services. We therefore suggest that healthcare systems more widely could benefit from adopting HSSs, to incorporate enhanced genetic analysis in patients in whom routine WGS analysis reveals NPF. While appropriate groups with such expertise may not be available in all healthcare systems, we have recently shown that international collaboration can facilitate this approach [e.g. The International Centre for Genomic Medicine in Neuromuscular Diseases (ICGNMD) advisory specialist MDTs]^19^. Separately, although machine learning and bioinformatic advances are improving automation of diagnostics, complex diagnoses remain elusive. Consequently, a personalised approach for such patients will be required for the foreseeable future.

One example of integrated data and clinical analysis in the UK is the rapid exome sequencing service for unwell children^20^. In this model, an expert team of clinical scientists work alongside a national network of clinical geneticists to provide urgent diagnoses for children with Mendelian diseases, supporting the case for specialised hub-and-spoke models. The delivery of an effective clinical genomic medicine service has major implications for healthcare systems and research worldwide; while we recognise the financial and time resource constraints within national health systems, it remains crucial that the diagnostic gains available from these data are maximised in the interests of patient care and science alike.

## Methods

### Standard approach

The research presented in this article complies with all relevant ethical regulations. Written informed consent was obtained from all participants or their guardians. The 100kGP was approved by the relevant Research Ethics Committee (REC) [East of England—Cambridge South (REC ref. 14/EE/1112)] and all participants provided informed consent. The present sub-study was undertaken as part of the Medical Research Council (UK) International Centre for Genomic Medicine in Neuromuscular Diseases (ICGNMD), which was approved by the relevant REC [London—Camberwell St Giles Research Ethics Committee (REC ref. 19/LO/1796)]. All participants in our sub-study provided informed consent. The patients presented in this study were referred with suspected PMDs and had common genetic causes for PMD excluded (recurrent single nucleotide variants in mtDNA and commonly affected mitochondrial DNA maintenance genes in nDNA) prior to WGS. WGS was undertaken on blood-extracted DNA (Illumina TruSeq, HiSeq 2500) via the 100kGP. HPO terms were extracted from physician notes and submitted by a non-physician healthcare professional during recruitment. Based on these phenotypic data, virtual gene panes were applied and the semantic similarity prioritisation tool ‘Exomiser’ was applied^21^. Standardised virtual gene panels were developed through the crowdsourced and curated resource ‘PanelApp’^22^. ‘PanelApp’ divides the status of genes into ‘green’ (diagnostic grade genes), ‘amber’ (genes with borderline evidence), and ‘red’ (genes with insufficient evidence). Only ‘green’ genes were included in clinical analysis. Resultant variants were prioritised into tiers. A clinical scientist reviewed all tier one (loss-of-function variants and other de novo protein altering variants in virtual panels applied) and tier two (non-loss-of-function protein altering variants in virtual panels applied, e.g. inherited missense variants). Possible variants were then discussed within an MDT environment. When there was consensus regarding the pathogenicity of a variant [American College of Medical Genetics and Genomics (ACMG) class IV ‘likely pathogenic or V ‘pathogenic’] it was confirmed with Sanger sequencing and reported, otherwise a report stating there were NPF was issued.

### Expanded approach

For all NPF cases a genomic medicine clinician reassessed the case. The phenotype (including HPO terms used during assessment) and ‘affected’ status in family members were reviewed. Modified Nijmegen mitochondrial disease diagnostic criteria were applied (see Supplementary Data)^23^. The genomic medicine clinician determined whether additional panels were required based on the phenotype, and whether the correct mode of inheritance had been considered according to the family history. All variants of uncertain significance (VUSs) identified in the standard approach were reassessed during the analysis to determine if additional clinical or literature data could upgrade their pathogenicity. Data was then reannotated by the bioinformatician in a clinical environment. Gene panels were expanded to include non-diagnostic grade (‘amber’ and ‘red’) genes and, when appropriate, additional gene panels were applied to investigate other aspects of the phenotype, omitted during the initial analysis. We annotated variants with Combined Annotation Depletion (CADD) scores, for protein coding variants, and Splice AI scores, for splicing variants^24,25^. When a panel revealed a coding variant in a phenotypically relevant recessive gene, analysis for a in trans non-coding variant was undertaken. In addition, copy number variant and structural variants generated by 100kGP using ‘Manta’ and ‘Canvas’ callers were analysed and prioritised using custom scripts^26^. We reviewed all CNVs overlapping coding sequences in the panels applied using ‘Manta’, for CNVs under 1Kb, and ‘Manta’ and ‘Canvas’, for CNVs over 1Kb. Finally, Mutect2, a somatic variant caller, was used to identify heteroplasmic variants in mtDNA^27^. In cases where a new diagnosis was identified, we used the clinician’s revised HPO terms and pedigrees to repeat Exomiser analysis and determine whether a more refined phenotype could achievethe same conclusion through a more automated approach.

### Validation of variants

For Patient E (*MYH2*) and Patient Q (*COX7B*) additional validation was undertaken.

#### Cell culture

Patient-derived fibroblasts (Patient Q—22y F) and three sex and age-matching healthy controls were grown in high glucose Dubecco’s Modified Eagle Medium (DMEM, ThermoFisher Scientific), supplemented with 4 mM glutamine, 110 mg/ml pyruvate, 10% (v/v) fetal bovine serum (Gibco, Life Technologies), 100 U/ml penicillin, and 100 mg/ml streptomycin (Gibco, Life Technologies). Cells were maintained at 37 °C under standard conditions (5% CO2; ambient O2; 95% relative humidity) and tested regularly for mycoplasma. Cells were collected by trypsinization, pelleted at 200 × *g* for 5 min, washed with PBS, and flash frozen in liquid nitrogen for later RNA preparation or protein extraction.

#### Western blot analysis

Cell pellets were lysed in RIPA buffer [50 mM Tris-HCl, pH 7.4, 150 mM NaCl, 0.25% sodium deoxycholate, 1 mM EDTA, 1% NP-40, 1X Complete protease inhibitor cocktail (Roche Molecular Diagnostics, Pleasanton, CA)]. Lysates were incubated on ice for 30 min and centrifugated at 12,000 × *g* for 15 min. Total protein extracts were resolved on a 10–12% Tricine polyacrylamide gel, transferred onto Trans-Blot nitrocellulose membrane (Bio-Rad), then incubated with primary antibodies against the following proteins: COX7B (ab137094, Abcam, 1:1,000 dilution); ATP5A, UQCRC2, COXII, SDHB, and NDUFB8 (ab110411, Abcam, 1:1,000 dilution); SDHA (14865-1-AP Proteintech, 1:1,000 dilution); ß-actin (4970, Cell Signalling, 1:10,000 dilution); and GAPDH (AM4300, ThermoFisher Scientific, 1:10,000 dilution), followed by Infrared dye labelled secondary antibodies. The following secondary antibodies were used: IRDye 800CW Goat anti-Mouse IgG (926–32210, Li-cor Biosciences, 1:10,000 dilution); IRDye 680LT Goat anti-Mouse IgG (926–68020, Li-cor Biosciences, 1:10,000 dilution); IRDye 800CW Goat anti-Rabbit IgG (926–32211, Li-cor Biosciences, 1:10,000 dilution); and IRDye® 680RD Goat anti-Rabbit IgG Secondary Antibody (926–68071, Li-cor Biosciences, 1:10,000 dilution). Images were detected with the Li-Cor Odyssey CLx infrared imager at 680 and 800 nm and normalised to ß-actin or GAPDH signal using ImageJ v.2.0.0 software (NIH, USA).

#### RNA isolation and gene expression analysis by qRT-PCR

To quantify gene expression by real-time quantitative polymerase chain reaction (qRT-PCR), total RNA was isolated from fibroblasts, using the RNeasy Mini Kit (Qiagen), and genomic DNA contamination was removed using DNA-free DNA Removal kit (ThermoFisher Scientific). RNA from human skeletal muscle biopsies (Patient E and three controls) was extracted using the RNeasy Fibrous Tissue Mini Kit (Qiagen). Quality of the extracted RNA was assessed by 1% agarose gel electrophoresis and from the A260nm/A280nm absorbance ratio (Nanodrop One, ThermoFisher Scientific). Next, cDNA was synthesised using the High-Capacity cDNA Kit (ThermoFisher Scientific). Finally, gene expression was determined using TaqMan Fast Advance Master Mix (ThermoFisher Scientific), according to manufacturer’s protocol, and qPCR reactions were undertaken using a QuantStudio 5 thermal cycler (ThermoFisher Scientific). All experiments were run in triplicate and the gene expression levels normalised to the B2M results using the ΔΔCq method^28^.

#### RT-PCR and Sanger sequencing

Confirmation of the *MYH2* c.4188-23T>A and *COX7B* c.40 + 5G>A variants was assessed using standard PCR-based sequencing. cDNA synthesised from skeletal muscle tissue (Patient E—25y F; *MYH2* c.4188-23T>A) or fibroblast (Patient Q—22y F; *COX7B* c.40 + 5G>A) RNA was amplified using Phusion Plus DNA Polymerase (ThermoFisher Scientific), according to manufacturer’s protocol. PCR product was run on a 1.2% (Patient E; *MYH2* c.4188-23T>A) or 2% (Patient Q; *COX7B* c.40 + 5G>A) agarose gel, isolated using Monarch DNA Gel Extraction Kit (New England BioLabs) and shipped for Sanger sequencing analysis (Genewiz). Primers are listed in the Oligonucleotides section.

#### Histochemistry and immunohistochemistry

Frozen sections from muscle biopsy samples (10 μm) were stained with haematoxylin and eosin, NADH tetrazolium reductase, or slow myosin heavy chain. Myosin developmental (NCL-MHCd, dilution 1:40) and Myosin neonatal (NCL-MHCn, dilution 1:40) antibodies were used. Myosin Heavy Chain antibody staining was performed on the Ventana Discovery Ultra (Roche) IHC platform, using the OmniMap anti-Ms HRP system (12 min at 36 C), followed by the Chromomap DAB Kit and counterstained with haematoxylin II (4 min). The working dilutions and the automated immunostaining protocols for antibodies against fast, slow, developmental (embryonic), and neonatal (fetal) myosin heavy chains (NCL-MHCf, NCL-MHCs, NCL-MHCd and NCL-MHCn) were performed in accordance with the optimised procedures used in the diagnostic muscle pathology laboratory (Dubowitz Neuromuscular Centre). The antibodies against Fast 2 A (7.5.2B, dilution 1:40) was obtained as a gift from Robin Fitzsimons and 2X (6H1, dilution 1:40) was obtained from DSHB, both of which were initially optimised in the diagnostic laboratory using a cohort of minimal change and dystrophic muscle biopsies. All experiments were performed by a senior histopathologist, who was blinded to all clinical data.

#### Statistical analysis

qPCR reactions were performed in technical triplicate, unless otherwise specified, from *n* = 3 or 5 biologically independent samples and reported as mean ± SEM. GraphPad Prism 8 software was used for data analyses (GraphPad Software Inc., CA). Figures were created using BioRender.com.

#### Oligonucleotides

All primers and probes used for *MYH2* c.4188-23T>A and *COX7B* c.40 + 5G>A qRT-PCR and PCR amplification were purchased from Integrated DNA Technologies (IDT) and are listed in our Supplementary Information, Supplementary Table 4. For OXPHOS transcript measurements, human probes were purchased from ThermoFisher Scientific and are also listed in our Supplementary Information, Supplementary Table 4.

### Reporting summary

Further information on research design is available in the Nature Research Reporting Summary linked to this article.

## Supplementary information

Supplementary Information

Reporting Summary

## Data Availability

The datasets analysed during the current study (primary data from the 100,000 Genomes Project) are subject to controlled access as they contain clinical information and the study’s ethical approval dictates that all data access and analysis must occur within the designated secure access environment. No data can be copied and removed from that environment without being subject to oversight from the study to ensure patient anonymity and data security. Data can be accessed worldwide by registered researchers who become members of a relevant Genomics-England Clinical Interpretation Partnership domain via the following link. https://www.genomicsengland.co.uk/about-gecip/for-gecip-members/data-and-data-access Applications will be reviewed by the appropriate domain lead within 10 working days, the researchers own institution must validate their affiliation and the researcher must complete a short online information training package. The current study did not generate novel custom code, rather it uses generically available scripts as indicated in the main text. 100,000 Genomes data is processed according to a generic pipeline described herehttps://research-help.genomicsengland.co.uk/display/GERE/10.+Further+reading+and+documentation?preview = /38047056/38047846/Genomics%20England%20Rare%20Disease%20Results%20Guide.pdf. All data supporting the findings described in this paper are available in the article and in the Supplementary Information and from the corresponding author upon reasonable request. Source data are provided with this paper.

## Source data

Source data
