# Supplementary material for: Specialist multidisciplinary input maximises rare disease diagnoses from whole genome sequencing
Source: Nat Commun. 2022 Nov 7;13:6324. doi: 10.1038/s41467-022-32908-7 (PMC9640711; doi:10.1038/s41467-022-32908-7)
Supplement: Supplementary file 1 — Supplementary Information [file 41467_2022_32908_MOESM1_ESM.pdf]

Supplementary Information

Supplementary Table 1: Current standard sequential workup for adults with suspected primary mitochondrial disorders (not including Leber hereditary optic neuropathy).

**Initial screening**
• Screening for common single nucleotide variants in blood and urothelial cells – m.3243A>G in *MT-TL1,* m.8344A>G in *MT-TK* and m.8993T>G/C in *MT-ATP6*<br>• Blood testing of CK, lactate, FGF-21 and white cell ubiquinone levels.<br>• Deep NGS sequencing of full mitochondrial genome from single amplicon long range PCR enriched blood-extracted DNA<br>• Virtual gene panel (22 genes) 'Mitochondrial DNA (mtDNA) maintenance' (R352) via Twist clinical exome *ABAT, AFG3L2, DGUOK, DNA2, DNM2, FBXL4, MFN2, MGME1, MPV17, OPA1, POLG, POLG2, RNASEH1, RRM2B, SLC25A4, SPG7, SUCLA2, SUCLG1, TK2, TOP3A, TWNK, TYMP* https://nhsgms-panelapp.genomicsengland.co.uk/panels/533/v1.2<br>• For younger patients, long-range PCR based analysis for large scale rearrangements in blood DNA (our centre typically undertakes this for young patients <20y)
**Advanced testing**
• Muscle biopsy (where patient consents) with histopathological analysis for mitochondrial changes and spectophotometric respiratory chain enzyme analysis<br>• Deep NGS sequencing of full mitochondrial genome from a single amplicon long-range PCR enriched muscle-extracted DNA and long-range PCR based analysis for large scale mtDNA rearrangements from muscle-extracted DNA.<br>• Digital droplet PCR analysis for muscle-extracted mtDNA depletion (our centre typically undertakes this for young patients <20y).<br>• Virtual gene panel (214 genes) 'Possible mitochondrial disorder - nuclear genes' (R63) https://nhsgms-panelapp.genomicsengland.co.uk/panels/539/v1.17 via Twist clinical exome.

Abbreviations: CK, creatine kinase; FGF-21, fibroblast growth factor 2; NGS, next generation sequencing.

Supplementary Table 2: Modified 'Nijmegen' Mitochondrial Disease Criteria[1–3]

**Clinical features**		**Scoring**
Muscular	Myopathy<br>Abnormal EMG<br>Motor developmental delay<br>Exercise intolerance	Maximal score for muscle is 2
Neurological	Developmental delay or ID<br>Speech delay<br>Dystonia<br>Ataxia<br>Spasticity<br>Neuropathy<br>Seizures or encephalopathy	Maximal score for neuro is 2
Multisystem	Any gastrointestinal tract disease<br>Growth delay or failure to thrive<br>Endocrine<br>Immune<br>Eye (vision) or Hearing<br>Renal tubular acidosis<br>Cardiomyopathy	Maximal score for multisystem 3
**Total clinical**		**Total clinical score is maximal 4**
**Metabolic/Radiological features**		**Scoring**
Metabolic	Lactate high at least 2x: (score 2)<br>Alanine high at least 2x<br>Krebs cycle intermediates<br>Ethylmalonic and methylmalonic acid)<br>3-methylglutaconic acid<br>CSF lactate, alanine	
Imaging /other	Leigh disease (score 2)<br>Stroke like episodes (score 2)<br>Basal ganglia/brain stem<br>Lactate peak on MRS<br>Leukoencephalopathy with brainstem and spinal cord involvement<br>Cavitating leukoencephalopathy<br>Leucoencephalopathy with thalamus involvement<br>Deep cerebral white matter involvement and corpus callosum agenesis	
**Total metabolic and radiological**		**Total metabolic & MRI is maximal 4**
**Sub-total (if no muscle biopsy)**		**Total score without histology is maximal 8**

Muscle biopsy features		Scoring
Morphology	Ragged red/blue fibers (score 4)<br>COX-negative fibers (score 4)<br>Reduced COX staining (score 4)<br>Reduced SDH staining<br>SDH positive blood vessels (score 2)<br>Abnormal mitochondria on EM (score 2)	
RCEA	Decreased RCEA (Score 2)	
**Total muscle biopsy**		Total for muscle biopsy is maximal 4
**Total MDC score**		**Total score is maximal 12**

*Similar to Riley *et al* we included decreased RCEA as an additional criterion though did not subdivide scores into degrees of abnormality. [3] We define normal RCEA ratios as follows: muscle RCE complex I 0.104-0.268; muscle RCE complex II+III 0.040-0.204; muscle RCE complex IV 0.014-0.034. Every element scores 1 unless indicated differently. The severity of each finding is not considered due to the progressive nature of the disease. A score of 1 indicates unlikely mitochondrial disorder, score 2-4: possible mitochondrial disorder, score 5-7 probable mitochondrial disorder and score ≥8 definite mitochondrial disorder. Abbreviations: COX, Cytochrome c oxidase; EMG, electromyography; L/P, lactate/pyruvate; RCEA, respiratory chain enzyme activity; SDH, succinate dehydrogenase.

SUPPLEMENTARY NOTES ON NEW DIAGNOSES

Nuclear DNA

American College of Medical Genetics and Genomics variant interpretation criteria (UK Association for Clinical Genomics specifications) were used to assess pathogenicity of variants. These are denoted in square brackets after variant names (III = variant of uncertain significance, IV = likely pathogenic, V = pathogenic). https://www.acgs.uk.com/quality/best-practice-guidelines/

MCOLN1 diagnosis (MIM # 252650), Patients A and B (siblings)

MCOLN1 (NM_020533.3) c.1207C>T p.(Arg403Cys) [IV] (NM_020533.3) c.681-19A>C [IV]

Patient A

50y F (female) born following a normal pregnancy to non-consanguineous parents. She was noted to be 'floppy' in the first few weeks of life and had a tremor at age three months. She sat at 8m and crawled at 10m. She walked at age 5y and was able to walk with a walking aid until her early 30s. She required glasses from early childhood. She developed spasticity and dystonia during late childhood. Scoliosis was noted in adolescence. Menarche was delayed at age 17y. Optic nerve atrophy was diagnosed in childhood, her vision declined in adulthood, and retinitis pigmentosa was diagnosed at age 33y. She had prominent drooling from infancy and had a severe reaction to Botulinum toxin administration for this in later life with profound dysphagia and resulted in recurrent aspiration pneumonia.[4] PEG was inserted at age 39 years for dysphagia. Benign gastric polyps have been noted in late forties and are under surveillance. A muscle biopsy was not undertaken. Research whole exome sequencing did not reveal a diagnosis.

Patient B

44y M (male) born at term with normal initial milestones and not hypotonic. (Sat at 6 months, crawled at 9 months and walked at 12 months and normal initial speech development). He required glasses from early childhood. He had childhood asthma. He developed dystonia and scoliosis in late childhood and spasticity in his 20s. He had a hand tremor which worsened in his mid-20s meaning he could no longer use feeding utensils. He was diagnosed with retinitis pigmentosa at age 31y. Similar to his sister he developed severe dysphagia after Botulinum toxin treatment for hypersalivation. A PEG was placed at age 38y for hydration support and

benign gastric polyps were noted at this age. A gastric tumour (adenocarcinoma) was noted 3 years later. It was managed conservatively with endoscopic resection on two occasions, and he passed away four years later. Sequencing of mtDNA in muscle was normal and research whole exome sequencing did not reveal a diagnosis. Complex II was reduced on muscle biopsy. (NADH ubiquinone reductase, 0.141 (normal, 0.104 to 0.268); succinate cytochrome C reductase, 0.019 (0.040 to 0.204); cytochrome oxidase, 0.014 (0.014 to 0.34)).

Factors contributing to diagnosis.

Both patients had very extensive metabolic work ups, including normal white cell lysosomal enzyme activities. RCEA demonstrated complex II+III chain deficiencies on spectrophotometric analysis, but no other biochemical abnormality was identified. Following review of the phenotype, a metabolic disease virtual panel was added to the analysis despite normal biochemical results, acknowledging that respiratory chain complex deficiencies can be a secondary affect from other metabolic diseases outside of PMD. A single heterozygous variant in *MCOLN1* was identified. Recognition of the strong phenotypic match between the patients and Mucolipidosis IV fuelled further evaluation of the non-coding space which identified a non-coding variant which had previously been described in a patient with suspected mitochondrial disease giving rise to intron retention, leading to introduction of a nonsense codon.[5] The variants were confirmed in both affected siblings and heterozygous parents.

These findings end a 50y search for a formal diagnosis for this family. Mucolipidosis type IV is technically a lysosomal storage disorder, however it is not associated with abnormal white cell enzyme activities, explaining the relatively unremarkable biochemical findings in this family, and emphasising the need for a molecular approach to diagnosis for these patients. While *MCOLN1* (a *TRPML1*) is technically a lysosomal calcium channel it is also physically involved in mitochondrial calcium regulation during mitochondrial-lysosomal contact. Patients with Mucolipidosis IV have abnormal mitochondrial-lysosomal contact and contact-dependant calcium uptake.[6] This suggests that this disorder might be categorised as both a lysosomal disease and PMD given that mutations in this channel directly affect the function of both organelles.

Mucolipidosis IV is associated with achlorhydria and elevated serum gastrin. Interestingly the carcinogenic bacterium Helicobacter pylori targets *MCOLN1* through its virulence factor VacA causing defective endolysosomal trafficking and killing and defective lysosomes can be colonised by the bacteria allowing them to evade treatments. [7,8] Given the presence of gastric polyps in both patients, and the development of gastric cancer in the male patient this would suggest that there may be an increased risk of gastric neoplasia in these patients.

Implications for management

We suggest screening for gastric neoplasia in affected individuals.

***POLR3A* diagnosis (MIM #607694), Patients C and D (twin siblings)**

POLR3A (NM_007055.4):c.2617-1G>A [V] (NM_007055.4):c.1909+22G>A [IV]

Patient C

41y F who was originally referred to paediatrics by health visitor at age 18m with delayed walking and short stature. She was able to run and ride a bike but was slower than her peers. In early adulthood she was thought to have developed a very slowly progressive myopathy and suffered from muscle cramps. Her muscle weakness was fatigue-induced. *RYR1, SMN1, DOC7, SEPN1, N88K, TPM3, FHL1, CRYAB, DES ZASP, ANO5, RAPSN* and *CHRNE* testing were negative. There was no clinical response to myasthenia treatments. Muscle biopsy showed fibre size small type 2 fibres, and core-like areas. At age 33y she was noted to have developed absent reflexes, extensor plantar reflexes, ptosis and low tone in her lower limbs and had delayed motor conduction studies. A repeat muscle biopsy at age 34y showed low complex I 0.021 (0.104+/- 0.036) complex II 0.078 (0.145 +/- 0.047) complex III 0.367(0.554+/-0.345) Complex IV 0.410 (1.124+/-0.511) and a small number of COX -ve fibres. mtDNA sequencing and screening for large-scale rearrangements in muscle-extracted DNA were normal. Repeat MRI, which was initially normal, showed symmetric high signal changes in the midbrain, superior cerebellar peduncles reaching the dentate nuclei. The multisystem nature of the phenotype and the presence of respiratory chain enzyme deficiencies raised the possibility of PMD as the diagnosis.

Patient D

This 41y F presented with walking difficulties in her teenage years. She was diagnosed with a very mild proximal myopathy and later a sensory axonal neuropathy. She demonstrated a spastic paraparesis later in adulthood. MRI brain was initially normal but later showed minimal periventricular white matter changes around the right occipital region with normal MRI spine.

Factors contributing to diagnosis

Initial evaluation of the family history noted that the siblings' father had a heart transplant for cardiomyopathy which led to a suspicion that this was a dominant multisystem disorder. However, on re-evaluation by the genomic medicine clinician, this was felt to be unrelated to the daughters' phenotype and the mode of inheritance was presumed autosomal recessive. The presence of the second sister's neuropathy led to the inclusion of a hereditary neuropathy panel in re-evaluation of the case. A canonical splice site variant (-1) was identified in *POLR3A.* This prompted re-evaluation of the non-coding space for an *in trans* intronic variant and the identification of a +22 recurrent pathogenic variant.

Reverse phenotyping identified that patient C was also noted to have abnormal dentition with absent primary incisors and delayed puberty in childhood. These are classical features of the 4H syndrome (hypomyelination with hypogonadotropic hypogonadism and hypodontia) associated with *POLR3A*. Re-evaluation of the MRI brain in conjunction with the literature demonstrated characteristic superior peduncle involvement which is especially associated with the c.1909+22G>A variant seen in these patients.[9,10] These specific features allow application of the ACMG PP4 criterion.

This case further widens the phenotype of this disorder to include a childhood onset myopathy. *POLR3A* is a subunit of RNA polymerase III. The exact mechanism through which defective *POLR3A* resulted in low respiratory chain enzymes in this case is not clear. Mitochondria use their own RNA polymerase, *POLRMT.* However, as many components of the respiratory chain are encoded in the nucleus, defective transcription of these proteins may give rise to suboptimal oxidative phosphorylation.

Implications for management

Reproductive options now exist for these two young women, and they no longer require diabetes and cardiac screening.

MYH2 diagnosis (MIM # 605637), Patient E

MYH2 (NM_017534.5):c.30del p.(Phe10LeufsTer51) [IV] (NM_017534.6) c.4188-23T>A[IV]

This 25y F was noted to have reduced eye movements by her optician at age 21 years. She was referred to the mitochondrial service with the presumptive diagnosis of chronic progressive external ophthalmoplegia. On examination she was noted to have bilateral ophthalmoparesis with mild hip flexion weakness. mtDNA sequencing and large-scale rearrangement analysis in muscle-extracted DNA were normal. Single fibre EMG and myasthenia antibody testing were normal.

Factors contributing to the diagnosis

On re-evaluation of the phenotype, she was noted to have muscle histology more in keeping with a myopathy than PMD and hence evaluation focused on a congenital myopathy panel. A single loss of function coding variant in _MYH2_ was identified. _MYH2_ is associated with both dominant and recessive disease. However, the patient's phenotype was felt to be more in keeping with the recessive form of disease. Using 'spliceAI' annotation a second non-coding variant was identified as likely to have a pathogenic effect (delta score acceptor gain 0.99). We extracted RNA from the patient's frozen muscle sample and undertook qPCR and PCR and gel electrophoresis on cDNA. qPCR revealed a significant down regulation of _MYH2_ compared with controls as demonstrated in main Figure 1c. The variant is predicted to introduce an acceptor site resulting in the inclusion of 23 nucleotides between exons 30 and 31 leading to a new stop codon occurring 41 nucleotides after exon 30. Gel electrophoresis of PCR products revealed a faint amplicon in the patient compared with control. This band showed wild type exon 30-31 sequence (see below). Given the very low expression of _MYH2_ seen on qPCR (<1% of control) we predict that the majority of _MYH2_ mRNA undergoes nonsense mediated decay, and the faint wild-type band is the result of leaky splicing.

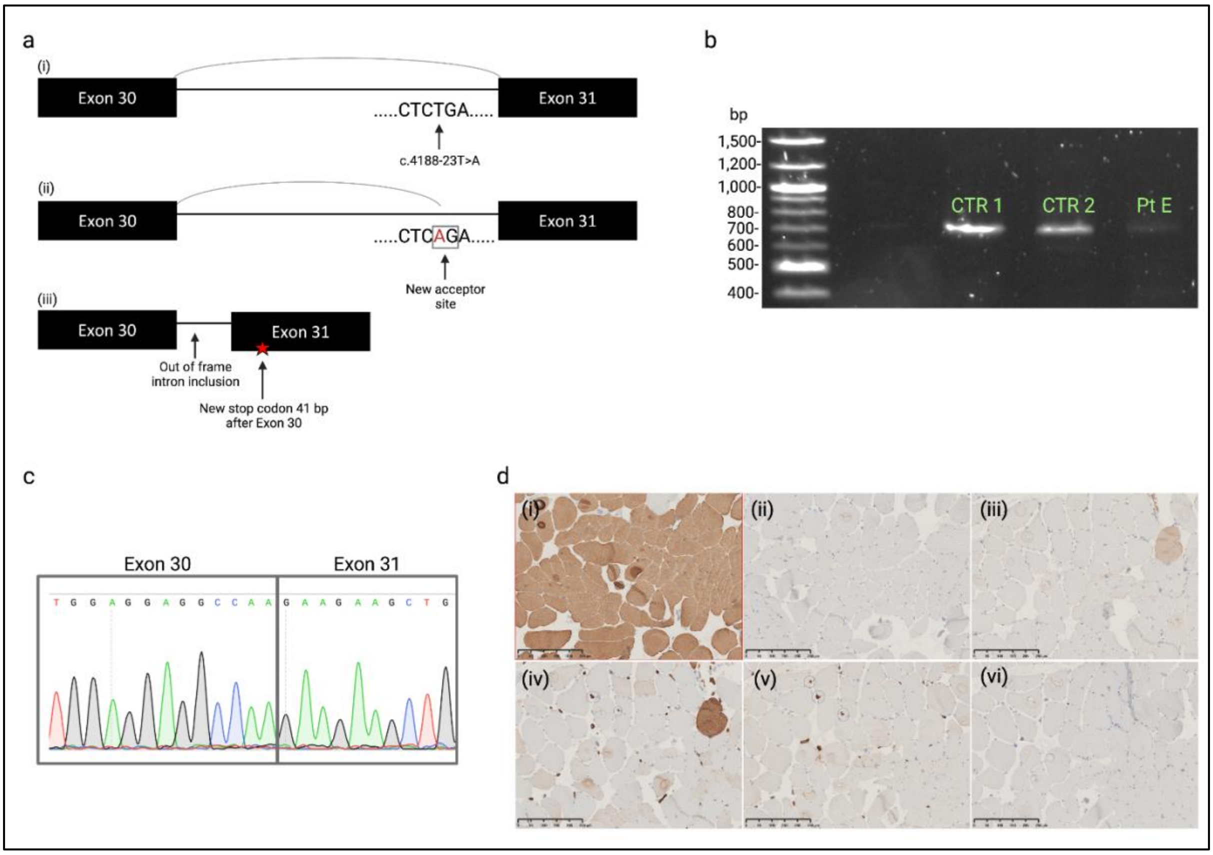

<u>Supplementary Figure 1</u>: Evidence for pathogenicity of novel non-coding variant in *MYH2*. a illustrates the intronic variant c.4188-23T>A (i) which is predicted to create a new acceptor site (ii) leading to an out of frame intronic inclusion and a resultant stop codon 41 bp after the end of exon 30 (iii). **b** shows gel electrophoresis of *MYH2* cDNA amplicons, the patient amplicon (Pt E) is extremely faint compared to two biologically independent controls (CTR 1 and CTR 2). The experiment was performed one time to confirm the qRT-PCR result shown in main Figure 2a (i). **c** Sequence electropherogram from cDNA of the patient E. The sequence is wild type indicating that while most of the transcript undergoes nonsense mediated decay, a small amount of transcript escapes the aberrant splicing event. **d** Immunostaining for myosin heavy chains showed marked slow fibre predominance supporting the biallelic loss of *MYH2*. Labelling for 2A fibre specific antibody '7.5.2B' was negative suggesting complete loss of 2A fibres [(i)= slow myosin, (ii)= 2A myosin (7.5.2B antibody), (iii)= 6H1 (2X myosin), (iv)=pan-fast myosin, (v)=fetal myosin and (vi)=embryonic myosin]. Each staining was performed in two serial sections.

This case highlights the importance of two factors. Firstly, without sufficient clinical input, the appropriate panels and inheritance patterns may not have been identified. A single loss of function allele may have been thought to fully explain the disease and resulted in inappropriate patient counselling. However, in depth appreciation of the patient's phenotype suggested this was the recessive form of this myopathy and prompted the identification of a second hit. Secondly, undertaking some simple functional studies was paramount in upgrading the non-coding variant from a class III VUS, to a likely pathogenic variant. This

highlights the need for inclusion of a translational scientist in a research or clinical laboratory to prove causation of pathogenic variants.

Implications for management

This young woman now has reproductive options and the diagnosis means that progression to severe disease is unlikely and cardiac screening can be rationalised as this is less likely than in PMD.

COXFA4 **(previously** ***NDUFA4*****) diagnosis (MIM # 619065), Patient F**

(ENST00000339600):c.131+1G>C homozygous [IV]

This 26y F was the product of normal pregnancy. She fed poorly in the neonatal period and developed poor swallow eventually requiring PEG tube. Motor milestones were delayed. She was diagnosed with Leigh syndrome at age 18m with abnormal MRI brain supporting the diagnosis. Ambulation deteriorated in early adulthood with pyramidal signs and spasticity.

Factors contributing to the diagnosis

This is a loss of function variant and disrupts a canonical splice site. This variant was not called by the routine approach as it does not include newly established genes – termed 'amber' or 'red' in the PanelApp system. Following advice from our centre this gene was upgraded to green status.

This diagnosis highlights the need for continuous input from clinicians regarding the content of gene panels as evidence in the literature changes. In the current era new morbid genes are established constantly and clinical testing must balance the inclusion of newly established genes and the availability of sufficient evidence to support their pathogenicity.

Implications for management

The diagnosis of a PMD supports the need for systemic screening for cardiomyopathy and arrhythmia, diabetes, deafness and renal dysfunction.

***COL4A2* diagnosis (MIM # 614483), Patient G**

(NM_001846.4): c.3736C>T p.(Gln1246Ter) [IV]

This 32y old F was born via emergency caesarean section for fetal distress. Deafness was noted in the neonatal period and attributed to perinatal difficulties. She developed diabetes at age nine years and seizures (visual abnormalities, followed by secondary generalised tonic-clonic seizures, treated with lamotrigine) in her teenage years. These resolved in her 20s. MRI brain showed occipitoparietal subcortical white matter changes. She had slightly elevated lactate 2.5 mmol/L (0.7-2.1) but normal muscle biopsy histology. She was also diagnosed with migraines, postural orthostatic tachycardia syndrome and developed severe fatigue in adulthood. Respiratory chain enzyme analysis, mtDNA sequencing and screening for large-scale rearrangements in muscle were normal.

<u>Factors contributing to the diagnosis</u>

The spectrum of disease associated with the *COL4* genes has expanded from porencephaly due to missense variants to milder disease such as subtler white matter changes and epilepsy[11]. In addition, variable penetrance and both missense and loss of function variants have now been reported.[12] The variant was not absent but extremely rare on gnomAD (allele frequency 0.000007205) and absent from neurogenetic patients, in keeping with this variable penetrance and disease severity. We arranged for the patient's neuroimaging to be reviewed by a clinician with expertise in epilepsy related to the *COL4* genes and the neuroimaging was in keeping with the disease spectrum.

This case highlights that reanalysis of genomic data should be accompanied by a thorough literature review to identify reports of expanding phenotypes. In the genomic medicine era the phenotypes associated with many genes continue to expand rapidly. This variant could have been dismissed if the wide phenotype associated with this gene, and its variable penetrance was not appreciated. While this finding is unlikely to explain the patient's diabetes, type 1 diabetes is of course a common disease of childhood and is typically not monogenic. This emphasises the problems encountered in patients presenting with mitochondrial phenotypes, in that they can have compound phenocopies of mitochondrial disease (two or more genetic or acquired diseases coming together to resemble a mitochondrial disorder).

Implications for management

This young woman now has reproductive options. In addition, screening for PMD complications can be stepped down.

***KCNQ4* Diagnosis (# 600101), Patient H**

(NM_004700.4):c.296_304dupTCGTCTACC p.(Tyr101_His102insLeuValTyr) [IV]

This 77y old M developed childhood-onset hearing loss. In his 60s he developed balance and movement problems and had choreiform movements of his hands. On further investigation he was found to have bilateral vestibular hypofunction and a moderate cerebellar syndrome, mild length dependant axonal sensory polyneuropathy, and cataracts. MRI brain was normal. Testing for Huntington disease and Huntington phenocopies, SCAs, CANVAS (cerebellar ataxia with neuropathy and vestibular areflexia syndrome) and ataxia telangiectasia was normal. He was referred for mitochondrial investigation given the combination of deafness and CNS disease with normal genetic work up and complex unusual family history with multiple family members with childhood or adult-onset deafness, and a history of cardiomyopathy in his brother and father. Normal large-scale rearrangements and mtDNA sequencing in muscle extracted DNA. Dominant family history of childhood-onset deafness.

Factors contributing to the diagnosis

In this case the mode of inheritance was reinterpreted by the clinician as autosomal dominant, with deafness as the central phenotype. This led to the discovery of a small indel in *KCNQ4.* This segregated in three affected family members; however, there was insufficient evidence to classify the variant as pathogenic or likely pathogenic. Following feedback from the clinical scientist the clinician arranged testing on many affected and unaffected family members. This supplied sufficient segregation data to apply ACMG criterion PP1 and upgrade the variant.

Accurate interpretation of pedigrees can be challenging in complex phenotypes. This case highlights the importance of pedigree interpretation for segregation data. While relatively simple, counselling family members, obtaining samples and undertaking Sanger confirmation of variants is time consuming for the clinician and scientist alike and this should be factored into workforce planning. We feel this family is likely to have a yet undetermined second

mutation and are undertaking research studies to identify this. We also suggest that in suspected mitochondrial disease phenotypes there is value in considering common genetic causes for parts of a presentation e.g., non-syndromic hearing loss, a relatively common disease.

Implications for management

The wider family now has options for reproductive planning including several family members of childbearing age.

***DNM2* diagnosis (MIM # 160150), Patient I**

(ENST00000355667.11):c.1810G>A p.(Glu604Lys) [IV]

This 25y F was born following a normal pregnancy. She was noted to be floppy at birth, and her presentation was initially thought to be in keeping with congenital myasthenic syndrome though this diagnosis was later revised. She sat at 14 months and walked at 20 months. She currently has bilateral ptosis and ophthalmoplegia, facial weakness, axial and limb weakness with proximal to distal gradient, elbow and finger contractures and stiff spine. Muscle biopsy demonstrated fibre type disproportion. (Supplementary Figure 2) She developed IBS (proctitis) in adulthood.

Factors contributing to this diagnosis

A mosaic variant in *DNM2* was identified in this case. This diagnosis was enabled by avoiding use of a hard cut off for variant calling. The variant was present in 16% of reads and was confirmed to be present on Sanger sequencing.

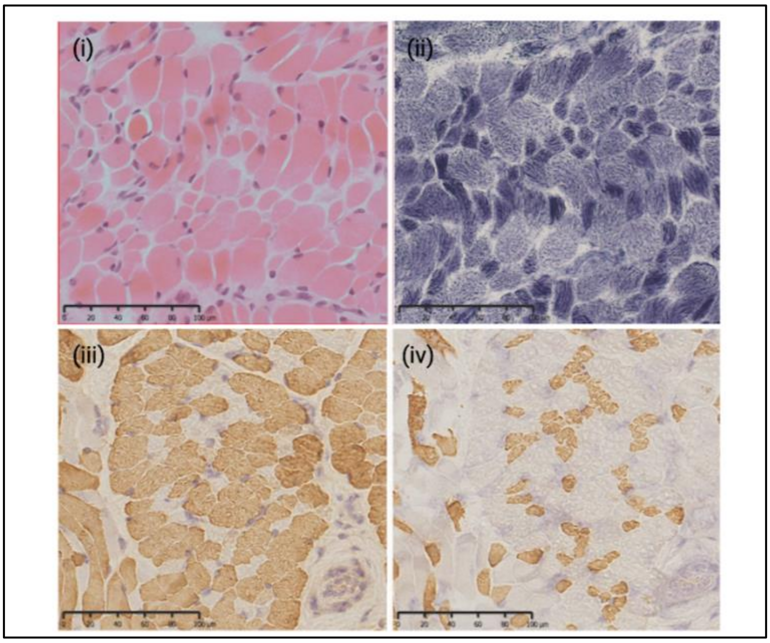

<u>Supplementary Figure 2</u>: Muscle biopsy Images from Patient I. Histopathology shows congenital fibre size disproportion, with mild overall fast fibre predominance; (i) Haematoxylin & eosin, (ii) NADHTR, (iii) fast myosin, and (iv) slow myosin with accompanying slow fibre predominance. Each staining was performed in two serial sections.

Dynamin 2 constitutes part of mitochondrial disorders, congenital myopathy, and neuropathy panels as it has been related to multiple mtDNA deletions, but the presentation primarily has a myopathy or Charcot Marie Tooth phenotype. This emphasises that many of the 'mitochondrial' defects identified on muscle biopsy (including mtDNA deletions and histological changes) are not pathognomonic for classical primary mitochondrial disease and a broad approach is needed to diagnosis. This mosaic variant was missed by the routine pipeline. As mosaic variants in myopathies are increasingly reported, *de novo* mosaic variants should be included in the analysis of these panels. This underlines the importance of involving a clinical bioinformatician in the MDT, ensuring pipelines are patient-focused.

<u>Implications for management</u>

A clinical trial for DYN101 (an antisense oligonucleotide) existed for this disease (https://www.clinicaltrials.gov/ct2/show/NCT04033159). The patient also has access to reproductive options. Screening for systemic complications for mitochondrial disease can be rationalised.

Mitochondrial DNA

While patients routinely have mtDNA sequenced as part of the diagnostic process, for historical reasons these variants may not have been recognised previously (e.g., earlier sequencing technology with insufficient depth to pick up lower level heteroplasmy, early primer position masking variants or insufficient evidence at time of sequencing to classify a variant as disease-causing.)

***MT-ND3* diagnosis (MIM # 500014), Patient J**

m.10158T>C (25% heteroplasmy) [IV]

31y F with a congenital cerebellar ataxia and learning disability. Her ataxia was stable until age 14y at which time her motor skills deteriorated. MRI demonstrated abnormal signal in the cerebellum, caudate nucleus, and putamen. Blood and CSF lactates were raised at 3mmol/L and 3.4mmol/L respectively. Muscle biopsy demonstrated a complex I deficiency 0.058 (0.104-0.206). In adulthood she developed severe epilepsy and recurrent stroke-like episodes. She has left sided spastic hemiparesis, optic disc atrophy and a right bundle branch block.

***MT-ATP6* diagnosis (MIM # 500015), Patient K**

m.8969G>A (85% heteroplasmy) [V]

28y M was born at term. The pregnancy was complicated by reduced fetal movements. He had head lag as an infant and he first walked at 3y. He has learning problems. He developed epilepsy at age 3 years. He has knee muscle contractures and severe constipation. MRI brain was normal. He has myopathic facies. Parents declined a muscle biopsy. The phenotypes associated with this mutation are very variable including mitochondrial myopathy, lactic acidosis and sideroblastic anaemia (MLASA) and IgG nephropathy. This patient does not have lactic acidosis, sideroblastic anaemia or nephropathy.

***MT- ATP6* diagnosis (MIM # 500015), Patient L**

m.8618dupT (29% heteroplasmy) [V]

44y F. The pregnancy was complicated by intrauterine growth restriction, and she had feeding difficulties and jaundice in the neonatal period. She walked at 18 months. She developed visual problems and was found to have a tapetoretinal disorder and optic atrophy. She also

developed deafness. She developed renal failure in adulthood requiring transplantation. Later in life she developed a progressive spastic paraparesis and supranuclear gaze palsy. Muscle biopsy demonstrated low complex II and III and pinprick vacuoles containing lipid. She was also diagnosed with diabetes in adulthood. Of note this case history was recently reported by our service.[13]

Factors contributing to these diagnoses

These diagnoses were made by applying a somatic variant caller to WGS data. PCR-free WGS can take advantage of the naturally high copy number of mtDNA present and achieve extremely deep >1,000x coverage of the mitochondrial genome for sensitive pick up of heteroplasmic variants. While these patients had mtDNA testing in advance of being enrolled for WGS, a diagnosis was not achieved for various reasons. (For example, use Sanger sequencing did not routinely identify low level mutations, suboptimal primer placement may have led to missed mutations, and insufficient evidence may have existed for to classify a variant as pathogenic). The current method using single-amplicon long-range PCR enrichment and deep NGS sequencing provides uniform deep coverage for more confident identification of variants.

These diagnoses highlight a potential role of WGS early in the diagnostic process for PMDs. Going forward we are likely to see increasing use of WGS for concurrent analysis of mtDNA and nDNA mitochondrial genes in diagnostic services. Interestingly, mtDNA genes may be included in virtual panels where PMD is rarely considered in the differential diagnosis. e.g. *Mt-TL1* in monogenic diabetes panels. It will be crucial that patients are consented appropriately for testing of mtDNA as the results may have very complex predictive implications for family members as we have discussed elsewhere. [14]

Implications for management

In addition to reproductive advice, as these patients now have confirmed mtDNA mutations we recommend routine screening for cardiomyopathy and arrhythmia, diabetes, deafness, and renal dysfunction. Patient K will also be eligible for a small molecule drug trial. (https://www.clinicaltrials.gov/ct2/show/NCT04535609)

Dual diagnoses

KARS1-LMNA dual diagnosis (MIM # 619196 and MIM # 150330), Patient M

KARS1 NM_001130089.1 c.683C>T p. (Pro228Leu) [IV] NM_001130089.1 c.774A>T p.(Arg258Ser) [IV]

LMNA NM_170707.3 c.667_668delGA p. (Glu223AspfsTer7) [IV]

Patient M is a 28y old female. Her birth history was normal. She became progressively hypotonic from around 3m of age and was less engaged with her surroundings. By 10m she still could not sit. Initial hearing test was normal, but she was diagnosed with sensorineural hearing loss and speech delay in childhood. At age 5y she developed myoclonic jerks, and seizures with absences. She has ataxia and uses a wheelchair for ambulation. Family history is significant for cardiomyopathy in her mother. Array CGH, mtDNA sequencing and rearrangement analysis did not reveal any abnormalities. Muscle biopsy at age 6y showed fibres with pale COX staining.

Factors contributing to the diagnosis

The variants in *KARS1* were picked up by the routine approach to data analysis, however, there was insufficient evidence for classifying c.774A>T variant as pathogenic. As with patient G (*COL4A2*) re-evaluation of the literature suggested that the phenotype associated with *KARS1* is wider than the initial cases described. The patient's presentation does not fit in neatly with any of the established phenotypes: Charcot Marie Tooth Intermediate B MIM # 613641, Recessive Deafness 89 MIM # 613916, Congenital deafness, and adult onset progressive leukoencephalopathy ('DEAPLE') MIM # 619196 or Progressive infantile leukoencephalopathy with or without deafness ('LEPID') MIM # 619147. We arranged for the patient's MRI Brain to be repeated to establish if a white matter change had developed and this was normal. Further interrogation of the recent literature established that the phenotype is now extremely broad and white matter changes are less of a cardinal feature. The *LMNA* variant is inherited from the patient's mother who has a mild cardiomyopathy.

This case highlights the existence of dual genetic diagnosis or 'double trouble'. The cardiomyopathy in this family is explained by a *LMNA* variant, but the syndromic patient's disease is from an unrelated genetic condition. Double trouble has been identified in 4.9% of

rare disease cases investigated via WES. [15] Clinicians and clinical scientists need to be attuned to this possibility and where appropriate interrogate data taking constituents of a phenotype as distinct entities with different genetic causes.

Implications for management

LMNA and *KARS1* carry a risk of cardiomyopathy and hence this screening is advisable for the proband. We also recommend vigilance for other complications of *KARS1* mutations e.g. neuropathy.

NSUN3 diagnosis (MIM # 619012), Patient N

(NM_022072.5):c.424C>T p.(Pro142Ser) homozygous [IV]

Patient N is a 26-year-old M. He was delivered at 41w via emergency caesarean section for oligohydramnios. Anaemia was first noted around 2 months of age, and he required transfusions from 2y 8m of age and was eventually diagnosed as having sideroblastic anaemia. In terms of development, he walked slightly late at age 18m. He is short, and growth hormone deficiency was diagnosed in childhood and he received GH injections and also has borderline hypothyroidism. He is microcephalic; his OFC 49.7cm (-3.6SD) centile MRI brain showed mild cerebellar hypoplasia in childhood but this was felt to be normal in adulthood. An axonal sensorimotor neuropathy was diagnosed in childhood and this is now severe with pes calvus, severe lower limb muscle wasting and clawing of fingers and toes. He was diagnosed with retinitis pigmentosa on electrodiagnostics age 14 years. Mild bilateral high frequency hearing loss was diagnosed age 20 years. Cardiology screening has revealed left ventricular non-compaction, bicuspid aortic valve, moderate aortic regurgitation, right sided aortic arch. Dysmorphology examination shows some facial asymmetry (presumably due to extramedullary haematopoiesis), plagiocephaly, low set ears, and a white patch in the hair over his occiput. Muscle biopsy age 13 years showed a COX deficiency (complex IV) 0.009 (0.014-0.034).

Factors contributing to diagnosis

NSUN3 had not reached the threshold for inclusion in clinical testing (amber gene) however, it was included in re-analysis leading to the identification of this variant. While his phenotype fits with features of Combined oxidative phosphorylation deficiency 48 (MIM # 619012) (e.g.,

microcephaly and COX deficiency) his developmental syndrome is less severe than previously published patients. *NSUN3* mediates methylation of cytosine to 5-methylcytosine (m5C) at position 34 of mt-tRNA(Met). Functional work was undertaken (data not shown, publication under review) and showed only background methylation indicating (1% of normal) indicating mt-tRNA(Met) is not being methylated in this patient.

Of note we believe that the patient is likely to have a dual 'double trouble' diagnosis and that is unlikely that the full spectrum of his syndrome is accounted for by the *NSUN3* variant. We have identified a second strong candidate gene, *CYB5D1*, involved in haeme binding that we believe to be the cause of his sideroblastic anaemia.

Implications for management

The result has reproductive implications for the patient and his siblings.

***MYCN* and *CAPN3* dual diagnosis (MIM # 164280 and MIM # 253600), Patients O and P**

CAPN3 (NM_000070.3) c.1504A>G (p.Ile502Val) homozygous [III]

MYCN (NM_005378.6) c.1181G>T (p.Arg394Leu) heterozygous [IV] (only present in Patient O)

Patient O is a 21-year-old F of consanguineous South Asian ancestry. At birth she had congenital lactic acidosis and was microcephalic. Cardiac investigation for lactic acidosis revealed a secundum ASD and small VSD. The ASD was treated with pericardial patching. Muscle biopsy demonstrated COX deficiency 0.006 (0.014-0.034) which resolved on repeat biopsy at age 12 years. mtDNA sequencing (including m.14674T>C) and depletion analysis were normal as was PDH activity in fibroblasts and white cell ubiquinone levels. Histology on her initial biopsy was normal, the repeat biopsy demonstrated excess lipid for age. Array CGH and karyotype were normal. Serum lactate normalised during childhood. She has good speech and normal hearing. She has an intellectual disability and severe ADHD. In recent years she complains of muscle ache and has mild thigh weakness. MRI of lower limb muscles is normal. CK is very mildly elevated. Adult OFC is 50.5cm.

Patient P is a 19-year-old F and younger sister of Patient O. She was born at term. She was healthy in the neonatal period however she had seizures due to hypocalcaemia and was found

to have a renal tubulopathy. Muscle biopsy demonstrated a complex IV deficiency 0.003 (0.014-0.034) and histology was within normal limits. She also had skin biopsy for fibroblast culture that showed normalised COX activity later in childhood. Plasma amino acids, urine organic acids, transferrin isoelectric focussing, white cell ubiquinone and very long fatty acid were normal. She has abnormal dentition. She has hearing problems due to chronic otitis media. She is non-dysmorphic. She has an unexplained recurrent neutropenia. Her learning level is normal.

<u>Factors contributing to diagnosis</u>

Re-evaluation of the phenotype demonstrated that the phenotype in the two sisters is very divergent. While the elder sister's (Patient O's) phenotype includes microcephaly, dysmorphic facial features and significant behavioural abnormalities, her sister, Patient P, has a uniform metabolic/ mitochondrial flavour. Given this interpretation the mode of inheritance filtering was widened to include a *de novo* dominant condition in the elder sister, and a shared recessive condition in both. This demonstrated a previously reported variant in *MYCN,* the cause of Feingold Syndrome 1. This variant is previously reported as likely pathogenic. Reverse phenotyping demonstrated that Patient O has typical features for Feingold Syndrome, absent in patient P. These include microcephaly, toe syndactyly, finger shortening and clinodactyly and deep-set eyes with short palpebral fissures. Interestingly when a limb girdle muscular dystrophy panel was added to investigate patient O's thigh pain and weakness, a homozygous variant in *CAPN3* was identified. While this has previously published as likely pathogenic, we classified it as a variant of uncertain significance. Notably the muscle biopsy performed in childhood was normal in both sisters and the MRI lower limbs in patient N in adulthood was within normal limits. The penetrance of *CAPN3*-limb girdle dystrophy is age dependant and has been reported above 40 years. A different amino acid substitution at this locus has been reported as pathogenic in compound heterozygous status with age of presentation up to 26y.[16]

We have identified a potential case of 'double-trouble' in this family. However, the cause of the mitochondrial/ metabolic phenotype remains elusive and we suspect an additional as yet uncovered variant is responsible for this.

Implications for management

Establishing the *MYCN* variant as the cause of Patient O's neuro-disability is useful as this is unlikely to reoccur in siblings when having children, and the patient could qualify for reproductive options should she wish.

Candidate diagnoses – strong variants of uncertain significance

COX7B diagnosis (MIM # 300887), Patient Q

(NM_001866.2): c.40+5G>A [III]

This 22y F was born at term following a pregnancy complicated by a fetal arrhythmia. She had positional talipes at birth. Her motor milestones were delayed (walked at 1.5y). She developed cataplexy at age 2 years which continues to be problematic. She was noted to have hypermobility early in childhood and was diagnosed with an intellectual disability. She has microcephaly. She fatigues easily and has proximal lower limb weakness. Investigation has demonstrated a raised CSF lactate. MRI brain showed cerebellar hypoplasia. She also has a neuropathy with a neurogenic EMG and right foot deformity requiring surgery. Echocardiogram has shown a small ASD. Muscle biopsy showed non-specific myopathic changes. RCEA was within normal limits. She has a tremor and anisocoria. Exome testing including sequencing of *DNMT1* did not reveal a cause.

Factors contributing to working diagnosis

This variant was one of several VUSs identified through routine analysis of the patient's genome. *COX7B* has been linked to an X-linked dominant disorder 'linear skin defects with multiple congenital anomalies 2' (MIM # 300885) though the pathogenesis for this disorder is not established. Given that this is a mitochondrial gene (COX7B is thought to be a subunit of the MT-CO2 module) we chose to investigate this variant in more detail.[17] This specific variant has previously been reported as *de novo*, likely pathogenic on ClinVar. We contacted the diagnostic laboratory that reported this variant to ClinVar. The patient is a female with developmental delay, dystonia, ataxia, and atrophy of the vermis and cerebellar hemispheres. We considered this a very relevant phenotype similar to that of our patient and extending the presentation beyond the classical syndromic presentation. However, according to NHS use of the ACMG criteria there was not sufficient evidence to classify this variant as likely pathogenic. Splice prediction scores suggested pathogenicity via weakening of the donor site

in intron 1 as the +5 position is typically conserved in splicing (MaxEntScan Alt/Ref 5.535/9.597 dbscSNV scores >0.9). Skewed X-inactivation studies were not possible as PCR analysis of the androgen receptor locus used for this study showed a single sized allele and could not be used to differentiate the chromosome pair. To investigate possible pathogenicity further we amplified fibroblast *COX7B* cDNA via PCR (Supplementary Figure 3b). Surprisingly, we observed an increased amplification of *COX7B*, suggesting an upregulation of wild type *COX7B* expression (Supplementary Figure 3c). This observation was confirmed by qPCR (main figure, panel d). Western blot analysis showed normal expression of COX7B and other OXPHOS proteins (Supplementary Figure 3d). However, when we examined the steady-state transcript levels of mitochondrial and nuclear encoded genes we noted an upregulation of mtDNA transcripts, which was not observed in the nuclear encoded mitochondrial genes (Supplementary Figure 3e).

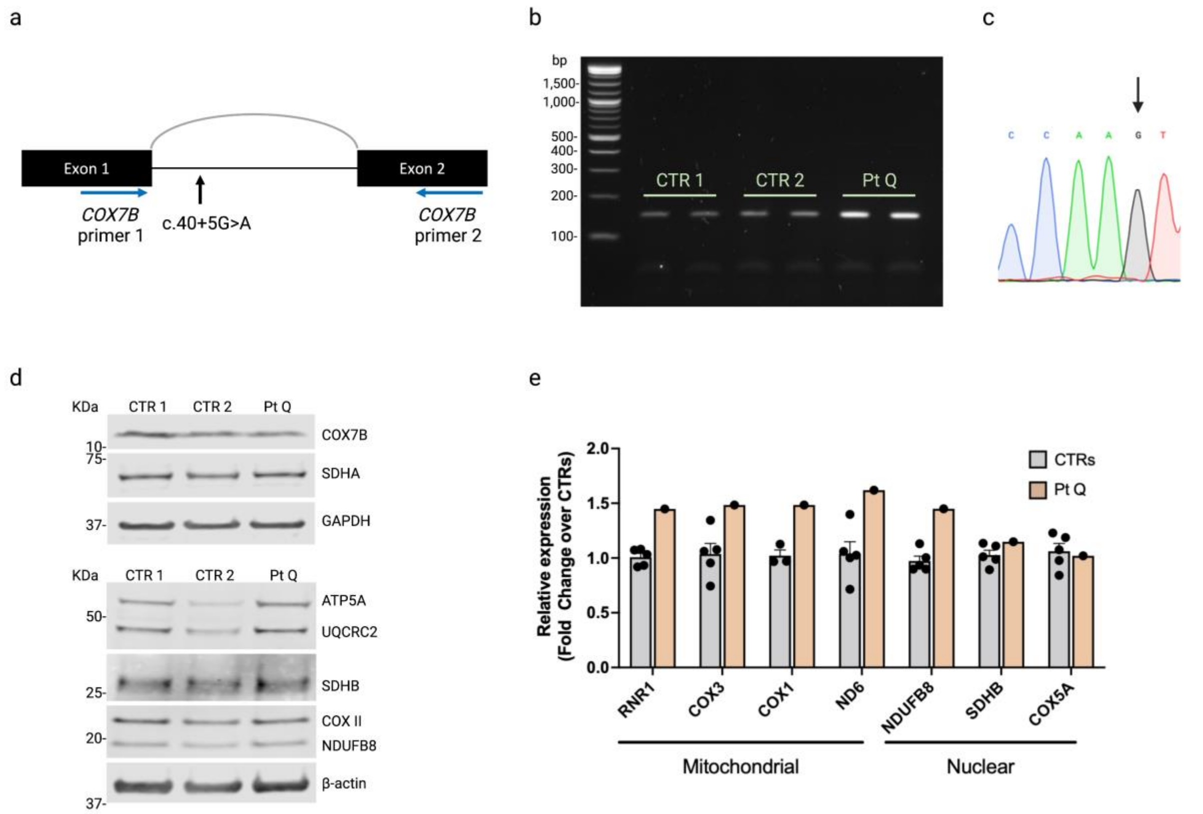

<u>Supplementary Figure 3</u>: Evidence suggesting pathogenicity of a splicing variant in *COX7b*. **a** illustrates the non-coding variant identified in *COX7B* and the position of the primers used to perform RT-PCR analysis. **b** Individual PCR of *COX7B* cDNA shows increased amplification of *COX7B* compared with two biologically independent controls (CTR1 and CTR2). Each PCR product was loaded twice. The experiment was performed one time to confirm the qRT-PCR result shown in main Figure 2b. **c** Sequence electropherogram from cDNA of Patient Q, showing that the upregulated amplicon is wild type. **d** Individual western blot analysis of protein lysates from two biologically independent controls (CTR 1 and CTR 2) and patient fibroblasts. **e** Steady-state transcript levels of mitochondrial and nuclear encoded genes analysed by qRT-PCR. Data are shown as mean values +/- SEM of n=5 biologically independent CTRs. COX1 data are shown as mean values +/- SEM of n=3 biologically independent CTRs. Each dot is the average of 4 independent measurements.

We speculate that the disease-causing mechanism of mutations in this gene is unlikely to be via a simple loss of function mechanism. Based on these findings we consider the variant to be a highly suspicious VUS but did not issue a diagnostic report despite it being reported as likely pathogenic in another centre. This underlines that even with a translational scientist in a diagnostic laboratory strong links with research are still necessary to establish novel disease mechanisms.

***KIF22* diagnosis (MIM # 603546), Patient R**

(NM_007317.3): c.377A>G p.(Tyr126Cys) [III]

This 29y F was born at 35 weeks due to preeclampsia. She was very hypotonic in the neonatal period. She had delayed motor skills sitting at 10m and walking at 18m and was always slow at sport. She had severe neonatal jaundice and was diagnosed with neonatal hepatitis with negative testing for genetic causes. She experiences severe fatigue. Her hands are small with proximally placed thumbs. Her fingers and toes are long and tapered and she has mild finger contractures. Her mid face is flat. She has mild generalised weakness which is non-progressive. CK and RCEA were normal. Muscle biopsy showed an increase in lipid content and a predominance of type 1 fibres.

Factors contributing to the working diagnosis

Given several unusual physical features the analysis was widened to include a skeletal dysplasia panel. This revealed a missense variant in *KIF22.* This gene is associated with spondyloepimetaphyseal dysplasia with joint laxity (Hall Type). Reverse phenotyping revealed velvety skin, and a history of recurrent dislocations. A skeletal survey was undertaken and showed long and slender fingers and elongated and slender femoral necks supporting a mild variant of the disease. Despite this strong phenotypic evidence, we could not upgrade the variant to likely pathogenic. As the patients' parents had passed away the variant could not be proven to be *de novo.* Additionally, the variant is extremely rare but not absent on gnomAD. However, the condition was known to have variable severity of penetrance among family members with minor changes being present in the father of an affected individual. [18] The variant is predicted pathogenic via a number of predictive tools including CADD (29), REVEL (0.944) and EVE (0.77).

This case highlights the need for extensive reverse phenotyping in some cases led by a genomic medicine clinician.

***NOXO1* diagnosis (Not MIM Morbid), Patient S**

(NM_172168.3):c.509dupG p.(Cys170TrpfsTer13)

This 21y F was the product of a normal pregnancy and her initial development was normal. At 2.5y she was noted to have visual problems and her vision deteriorated rapidly over 18

months: she is now registered blind. She was noted to have learning problems in childhood and has an intellectual disability. In later childhood she developed an auditory neuropathy spectrum disorder and cerebellar ataxia. MRI brain showed mild volume loss of the right hippocampus in comparison to the left. RCEA in muscle was normal.

Factors leading to the working diagnosis.

Our analysis included genes with non-clinical grade evidence on panels ('amber' and 'red genes'). This identified a homozygous variant in *NOXO1.* This gene is involved in the activation of NAPDH oxidases and is felt to be very important in otoconia formation. Knock out mice demonstrate balance problems rescued by re-introduction of *NOXO1.* [19]

Though this gene is not yet considered to have sufficient evidence for clinical use we feel this diagnosis here is secure. This is a homozygous loss of function variant in a gene known to cause a similar balance phenotype in a model organism. This diagnosis highlights that there is a need for clinicians to include genes with less definitive evidence in their diagnosis searches; otherwise, diagnoses will be missed. It also emphasises the need to develop links with translational scientists in clinical and research spaces to validate such findings functionally.

Supplementary Table 3: Patients diagnosed via routine and enhanced approaches

Routine diagnoses						
**ID**	**Clinical details**	**Nijmegen Score**	**Zygosity**	**Variant [ACMG Classification]**	**Disease**	**Notes**
1	46y M. Ptosis and weakness, multiple deletions	Probable (7)	Hom	*TK2* NM_004614.4 c.323C>T p.(Thr108Met) [V]	PMD	
2	24y F. Congenital muscular dystrophy with hyperCKemia, ataxia, tremor, sensory neuropathy, Learning disability	Probable (7)	Hom	*MICU1* NM_001195518.2 c.1078-1G>C [V]	PMD	
3	20y M. Demyelinating sensorimotor neuropathy, skeletal dysplasia, myopathy, complex IV deficiency, hypoglycaemia	Probable (6)	Hom	*COA3* NM_001040431.2 c.215A>G; p.(Tyr72Cys) [IV]	PMD	
4	18y M. ID, GDD, Tonic clonic seizures, myoclonic seizures,	Definite (9)	Hom	*SERAC1* NM_014363.5 c.127G>A p.(Gly43Arg) [IV]	PMD	
5	32y F. Motor predominant length dependant neuropathy, fatigue	Possible (3)	Homop	*MT-ATP6* m.9185 T>C [V]	PMD	
6	17y M. Short stature, spasticity, motor neuropathy, motor	Definite (10)	C Het	*C12orf65* NM_152269.4 c.210del p.(Gly72Alafs*13) [V] c.258_270dup p.(Ile91Hisfs*16) [IV]	PMD	

	neuropathy, grey matter abnormalities on MRI, severe visual impairment, squint					
7	18y F. Fatigue, pain, ptosis, anxiety, complex IV deficiency, Asperger's	Probable (6)	Het	*ZNF462* NM_021224.4 c.3831del p.(Tyr1278Metfs*3) [V]	NDD	
8	24y F. Fatigue, pain, ptosis, anxiety, complex IV deficiency, Asperger's	Probable (6)	Het	*ZNF462* NM_021224.4 c.3831del p.(Tyr1278Metfs*3) [V]	NDD	
9	33y M. Myoclonic epilepsy and non-epileptic attacks, right-sided episodes of weakness, Bilateral sensorineural hearing loss, Asperger's, bilateral basal ganglia calcifications, dyspraxia and dyslexia, low serum pyruvate, documented hypoglycaemic episodes	Probable (5)	Het	*CACNA1A* NM_001127221.1 c.2186_2187dup p.(Glu730Argfs*21) [IV]	Chan.	Partial diagnosis
10	30y F. Dystonia, ataxia, dysphagia (PEG in place)	Possible (5)	Het	*C19orf12* NM_001031726.2 c.278dup p.(Ala94Cysfs*8) [IV]	NBIA	
11	29y F. Ataxia and hearing impairment, cerebellar signs, mild learning difficulties	Probable (6)	C Het	*PNPLA6* NM_006702.4:c.2944_2947dup p.(Arg983GlnfsTer38) [V] NM_006702.4:c.721C>T p.(Arg241Trp) [IV]	SPG	
12	35y F. Muscular dystrophy, fatigue and cardiopathy	Possible (3)	Hom	*DES* NM_001927.3 c.364T>C p.(Tyr122His) [V]	M Dys.	

13	56y F. Myopathy, axonal peripheral neuropathy	Probable (7)	C Het	*GNE*<br>NM_001128227.2 c.1853T>C p.(Ile618Thr) [V]<br>NM_001128227.2 c.2179G>A p.(Val727met) [IV]	Myop.	
14	62y M. Myopathy, proximal weakness, COX -ve, RRF	Probable (7)	Hom	*GMPPB*<br>NM_013334.3 c.1081G>A p.(Asp361Asn) [IV]	MDys.	
15	61y F. Left ventricular non-compaction cardiomyopathy, family history of sudden cardiac death	Possible (3)	Het	*TNNT2*<br>NM_001276345.1 c.311G>T p.(Arg104Leu) [IV]	Cardio.	
16	61y M. Severe gastroparesis, cardiomyopathy, deafness, ptosis	Definite (9)	Het	*TTN*<br>NM_133378.4 c.52222+1G>A p.? [IV]	Cardio.	Partial diagnosis
17	44y M. Severe myalgia and cramps, muscle fatigue, calf hypertrophy, previously elevated CK, hearing loss	Probable (6)	Het	*KCNQ4*<br>NM_004700.3 c.961G>A p.(Gly321Ser) [IV]	Deaf.	Partial diagnosis
				**New diagnoses**		
**ID**	**Narrative**	**Nijmegen Score**	**Zygosity**	**Variant [ACMG Classification]**	**Disease**	**Notes**
A	50y F. Retinitis pigmentosa, deafness, drooling, dysphagia, drooling, somnolence, Complex II+III deficiencies	Definite (8)	C Het	*MCOLN1*<br>(NM_020533.3):c.1207C>T p.(Arg403Cys) [IV]<br>(NM_020533.3):c.681-19A>C<br>[IV]	LSD<br>SMD	Added metabolic panel, searched for non-coding second hit given strong

						phenotypic link to gene
B	44y M. Retinitis pigmentosa, dystonia, gastric cancer, urinary incontinence, dysphagia, severe fatigue, tremor, complex II + III deficiency, iron deficiency anaemia	Definite (8)	C Het	*MCOLN1* (NM_020533.3):c.1207C>T p.(Arg403Cys) (NM_020533.3):c.681-19A>C	MLIV SMD	Added metabolic panel, searched for non-coding second hit given strong phenotypic link to gene
C	41y F. Delayed motor milestones and mild myopathy diagnosed in early adulthood. In adulthood developed progressive CNS disorder: leukodystrophy, sensory impairment, gait apraxia, increased muscle fatiguability, abnormality of the brainstem white matter, abnormal pyramidal signs, Cytochrome C oxidase-negative muscle fibres, ptosis, dysarthria	Definite (9)	C Het	*POLR3A* *(*NM_007055.4):c.2617-1G>A [V] (NM_007055.4):c.1909+22G>A [IV]	LD	Analysed 2 sisters as same cause based on updated family history, added inherited neuropathy panel given predominance of this symptom in sib, searched for non-coding second hit given strong phenotypic link
D	41y F. Axonal neuropathy, mild proximal myopathy and	Probable (5)	C Het	*POLR3A* *(*NM_007055.4):c.2617-1G>A (NM_007055.4):c.1909+22G>A	LD	Analysed 2 sisters as same cause based on

	now increasing spastic paraparesis, early periventricular white matter changes					updated family history, Added inherited neuropathy panel given predominance of this symptom in this patient, searched for non-coding second hit given strong phenotypic link
E	25y F. Progressive External Ophthalmoplegia	Possible (2)	C Het	*MYH2* (NM_017534.5):c.30del p.(Phe10LeufsTer51) [IV] (NM_017534.6) c.4188-23T>A [IV]	Myop	Searched for non—coding second hit given specificity of gene's phenotype
F	26y F. Leigh syndrome	Definite (12)	Hom	*COXFA4* (ENST00000339600):c.131+1G>C [IV]	PMD	Included genes which had not yet achieved 'green' diagnostic grade on PanelAp

G	32y F. Migraine, Fatigue, epilepsy, hearing loss, diabetes, WM changes	Probable (6)	Het	*COL4A2* (NM_001846.4):c.3736C>T p.(Gln1246Ter) [IV]	NVD	Added broader epilepsy panel, new publications established gene is newly associated with variable phenotype, outside of porencephaly
H	77y M. Deafness, dystonia, neuropathy, vestibular hypofunction	Possible (3)	Het	*KCNQ4* (NM_004700.4):c.296_304dupTCGTCTACC p.(Tyr101_His102insLeuValTyr) [IV]	Deaf	Reinterpreted family history
I	25y F. Neonatal hypotonia, limited eye movements, elbow and finger contractures, axial, proximal and distal weakness, rigid spine	Possible (2)	Mosaic	*DNM2* (ENST00000355667.11):c.1810G>A p.(Glu604Lys) [IV]	Myop	Identified *de novo* mosaic variant with improved filtering strategy
J	31y F. Cerebellar ataxia, spastic hemiparesis, learning difficulties, optic disc atrophy, incomplete right bundle branch block, epilepsy	Probable (6)	25% Heterop	*MT-ND3* m.10158T>C [IV]	PMD	Improved filtering for heteroplasmic variants
K	28y M. Epilepsy and learning disability	Probable (7)	85% Heterop	*MT-ATP6* m.8969G>A [V]	PMD	Improved filtering for heteroplasmic variants

L	44y F. Early cataracts, thyroid dysfunction, renal dysfunction (transplant in 2006), diabetes, tapetoretinal disorder with optic atrophy (blind), deafness, cognitive decline, Asperger's, aortic regurgitation	Definite (8)	29% Heterop	*MT-ATP6* m.8618dupT [V]	PMD	Improved filtering for heteroplasmic variants
M	28y F. Regression, delayed motor milestones, SNHL, ataxia – gait and limb, myoclonus, epilepsy, family history of cardiomyoapthy	Probable (6)	C Het<br><br><br><br>Het	*KARS1*<br>NM_001130089.1 c.683C>T p.(Pro228Leu) [IV]<br>NM_001130089.1 c.774A>T p.(Arg258Ser) [IV]<br><br>*LMNA*<br>NM_170707.3 c.667_668delGA p.(Glu223AspfsTer7) [IV]	PMD<br><br><br><br>Cardio.	Literature has expanded phenotype and identified second variant in another individual
N	26y M. Mild learning problems, sideroblastic anaemia, large fibre sensory motor axonal polyneuropathy, mild high frequency sensorineural hearing loss, COX deficiency, dysmorphism	Probable (6)	Hom	*NSUN3* (NM_022072.5):c.424C>T p.(Pro142Ser) [IV]	PMD	Included genes which had not yet achieved 'green' diagnostic grade on PanelAp
O	21y F. Congenital lactic acidosis with reversible COX deficiency, dysmorphism, microcephaly,	Probable (7)	Het	*MYCN* (NM_005378.6) c.1181G>T p.(Arg394Leu) [IV]	NDD	Updated mode of inheritance filtering to consider intellectual

	intellectual disability, ADHD, muscle pain and mild proximal weakness					disability syndrome as distinct entity not in sister, reverse phenotyping in keeping with this syndrome
**Strong variants of unknown significance**						
**ID**	**Narrative**	**Nijmegen Score**	**Zygosity**	**Variant [ACMG Classification]**	**Disease**	**Notes**
O	21y F. Congenital lactic acidosis with reversible COX deficiency, dysmorphism, microcephaly, intellectual disability, ADHD, muscle pain and mild proximal weakness	Probable(7)	Hom	*CAPN3* (NM_000070.3) c.1504A>G p.(Ile502Val) [III]	MDys	Muscle pain and weakness may be explained by this variant
P	19y F. Reversible COX deficiency, history of hypocalcaemia and renal tubulopathy, chronic otitis media, neutropenia. No muscle symptoms.	Probable (7)	Hom	*CAPN3* (NM_000070.3) c.1504A>G p.(Ile502Val) [III]	MDys	Variant not yet penetrant in this patient
Q	22y F. Microcephaly, raised CSF lactate, exercise intolerance, cerebellar hypoplasia	Probable (7)	Het	*COX7B* (NM_001866.2):c.40+5G>A [III]	PMD	

R	29y F. Myopathy, fatigue, previous episode of encephalopathy, long tapered fingers with contractures | reverse phenotyping identified elongated femoral necks and finger bones, and a history of recurrent subluxations	Possible (3)	Het	*KIF22* (NM_007317.3): c.377A>G p.(Tyr126Cys) [III]	SD	Unusual facial appearance prompted inclusion of skeletal dysplasia panel; reverse phenotyping identified a hypomorphic form of Spondyloepimetaphyseal dysplasia with joint laxity type 2
S	21y F. Optic atrophy, cerebellar ataxia, learning problems, auditory neuropathy spectrum disorder	Possible (3)	Hom	*NOXO1* (NM_172168.3):c.509dupG p.(Cys170TrpfsTer13)	Deaf.	Only strong homozygous candidate – consanguineous background

Abbreviations: Cardio., Cardiomyopathy; Chan., Channelopathy; C Het, Compound heterozygous; COX-ve, Cytochrome C Oxidase negative muscle fibres; Deaf., Deafness; F, Female, Het, Heterozygous; Heterop = Heteroplasmic; Hom, Homozygous; Homop, Homoplasmic; LD, leukodystrophy, LSD; Lysosomal storage disease; M, Male, MDys., Muscular Dystrophy; MLIV, Mucolipidosis IV; Myop., Myopathy; NBIA, Neurodegeneration with brain iron accumulation; NDD, Neurodevelopmental Disorder; NVD, neurovascular disorder; PMD, Primary mitochondrial disease; RRFs, Ragged red fibres; SD, Skeletal dysplasia; SMD, secondary mitochondrial dysfunction; SPG, Spastic paraplegia; y, years

Supplementary Table 4: Primers and probes used for *MYH2* c.4188-23T>A and *COX7B* c.40+5G>A, and probes and catalogue numbers for OXPHOS subunits

Primer/Probe	Sequence (5'-3')	Comments
MYH2 primer 1	GGAGATAAAAGCCAAGAACGC	RT-PCR and Sanger sequencing
MYH2 primer 2	GTTCAAGAGATGCCTCTGCTTC	
COX7B primer 1	TTCACGATGTTTCCCTTGGT	
COX7B primer 2	GAAAGTGGCTCCACTAGCTAATAC	
MYH2 probe	56-FAM/AGCTCGGCC/ZEN/TTGGATTCCTGC	*qRT-PCR*
MYH2 primer 1	GCATGCCCTGCAGTCTT	
MYH2 primer 2	TGTTGGCCTTGGACAGTG	
COX7B probe	56-FAM/CAGAGCCAC/ZEN/CAGAAACGTACACCT	
COX7B primer 1	CCACTAGCTAATACAGCATTACCG	
COX7B primer 2	GTCAAAAGCGCACTAAATCGTC	
**Probe**	**Catalogue Number**	**Comments**
MT-ND6	Hs02596879_g1	Mitochondrial DNA encoded
MT-RNR1	Hs02596859_g1	
MT-CO3	Hs02596866_g1	
MT-CO1	Hs02596864_g1	
SDHB	Hs01042481_m1	Nuclear DNA encoded
COX5A	Hs00362067_m1	
B2M	Hs00187842_m1	

Supplementary Table 5: Comparison of patients diagnosed via our paper vs Schon et al.

**Comparison with Schon *et al.***

Number of overlapping patients with this paper	57/102
Number of overlapping patients in our newly diagnosed cohort	10/18
Number of newly diagnosed patients identified in Schon et al	3/10<br><br>All were mtDNA diagnoses, i.e., identified by an expanded bioinformatic approach (Patients J, K and L)
Number of newly diagnosed patients missed in Schon et al	7/10
Contributing factors to missed diagnosis	1. Missed nuance in family history (x5 diagnoses)<br>2. Missed nuance in clinical history (x2 diagnoses)<br>3. Missed second hit<br>4. Missed double trouble (x2 diagnoses)

Supplementary References

1. Morava, E. *et al.* Mitochondrial disease criteria: diagnostic applications in children. *Neurology* **67**, 1823–1826 (2006).
2. Witters, P. *et al.* Revisiting mitochondrial diagnostic criteria in the new era of genomics. *Genet. Med.* **20**, 444–451 (2018).
3. Riley, L. G. *et al.* The diagnostic utility of genome sequencing in a pediatric cohort with suspected mitochondrial disease. *Genet. Med.* **22**, 1254–1261 (2020).
4. Gioltzoglou, T., Cordivari, C., Lee, P. J., Hanna, M. G. & Lees, A. J. Problems with botulinum toxin treatment in mitochondrial cytopathy: case report and review of the literature. *J. Neurol. Neurosurg. Psychiatry* **76**, 1594–1596 (2005).
5. Kremer, L. S. *et al.* Genetic diagnosis of Mendelian disorders via RNA sequencing. *Nat. Commun.* **8**, 1–11 (2017).
6. Peng, W., Wong, Y. C. & Krainc, D. Mitochondria-lysosome contacts regulate mitochondrial Ca(2+) dynamics via lysosomal TRPML1. *Proc. Natl. Acad. Sci. U. S. A.* **117**, 19266–19275 (2020).
7. Capurro, M. I., Prashar, A. & Jones, N. L. MCOLN1/TRPML1 inhibition - a novel strategy used by Helicobacter pylori to escape autophagic killing and antibiotic eradication therapy in vivo. *Autophagy* **16**, 169–170 (2020).
8. Capurro, M. I. *et al.* VacA generates a protective intracellular reservoir for Helicobacter pylori that is eliminated by activation of the lysosomal calcium channel TRPML1. *Nat. Microbiol.* **4**, 1411–1423 (2019).
9. Harting, I. *et al.* POLR3A variants with striatal involvement and extrapyramidal movement disorder. *Neurogenetics* **21**, 121–133 (2020).
10. Minnerop, M. *et al.* Hypomorphic mutations in POLR3A are a frequent cause of sporadic and recessive spastic ataxia. *Brain* **140**, 1561–1578 (2017).
11. Zagaglia, S. *et al.* Neurologic phenotypes associated with COL4A1/2 mutations: Expanding the spectrum of disease. *Neurology* **91**, e2078–e2088 (2018).
12. McGovern, M., Flanagan, O., Lynch, B., Lynch, S. A. & Allen, N. M. Novel COL4A2 variant in a large pedigree: Consequences and dilemmas. *Clinical genetics* vol. 92 447–448 (2017).
13. Bugiardini, E. *et al.* Expanding the molecular and phenotypic spectrum of truncating MT-ATP6 mutations. *Neurol. Genet.* **6**, e381 (2020).

14. Macken, W. L., Lucassen, A. M., Hanna, M. G. & Pitceathly, R. D. S. Mitochondrial DNA variants in genomic data: diagnostic uplifts and predictive implications. *Nat. Rev. Genet.* **22**, 547–548 (2021).
15. Posey, J. E. *et al.* Resolution of disease phenotypes resulting from multilocus genomic variation. *N. Engl. J. Med.* **376**, 21–31 (2017).
16. Ten Dam, L. *et al.* Autosomal recessive limb-girdle and Miyoshi muscular dystrophies in the Netherlands: The clinical and molecular spectrum of 244 patients. *Clin. Genet.* **96**, 126–133 (2019).
17. Signes, A. & Fernandez-Vizarra, E. Assembly of mammalian oxidative phosphorylation complexes I-V and supercomplexes. *Essays Biochem.* **62**, 255–270 (2018).
18. Hall, C. M., Elcioglu, N. H., MacDermot, K. D., Offiah, A. C. & Winter, R. M. Spondyloepimetaphyseal dysplasia with multiple dislocations (Hall type): three further cases and evidence of autosomal dominant inheritance. *Journal of medical genetics* vol. 39 666–670 (2002).
19. Kiss, P. J. *et al.* Inactivation of NADPH oxidase organizer 1 results in severe imbalance. *Curr. Biol.* **16**, 208–213 (2006).
