# Supplementary material for: Specialist multidisciplinary input maximises rare disease diagnoses from whole genome sequencing
Source: Nat Commun. 2022 Nov 7;13:6324. doi: 10.1038/s41467-022-32908-7 (PMC9640711; doi:10.1038/s41467-022-32908-7)
Supplement: Supplementary file 2 — Reporting Summary [file 41467_2022_32908_MOESM2_ESM.pdf]

nature portfolio

Corresponding author(s): Robert DS Pitceathly, Jana Vandrovcova

Last updated by author(s): 2022/08/15

Reporting Summary

Nature Portfolio wishes to improve the reproducibility of the work that we publish. This form provides structure for consistency and transparency in reporting. For further information on Nature Portfolio policies, see our Editorial Policies and the Editorial Policy Checklist.

Statistics

For all statistical analyses, confirm that the following items are present in the figure legend, table legend, main text, or Methods section.

n/a	Confirmed	
☐	☒	The exact sample size (n) for each experimental group/condition, given as a discrete number and unit of measurement
☐	☒	A statement on whether measurements were taken from distinct samples or whether the same sample was measured repeatedly
☒	☐	The statistical test(s) used AND whether they are one- or two-sided<br>*Only common tests should be described solely by name; describe more complex techniques in the Methods section.*
☒	☐	A description of all covariates tested
☒	☐	A description of any assumptions or corrections, such as tests of normality and adjustment for multiple comparisons
☒	☐	A full description of the statistical parameters including central tendency (e.g. means) or other basic estimates (e.g. regression coefficient) AND variation (e.g. standard deviation) or associated estimates of uncertainty (e.g. confidence intervals)
☒	☐	For null hypothesis testing, the test statistic (e.g. F, t, r) with confidence intervals, effect sizes, degrees of freedom and P value noted<br>*Give P values as exact values whenever suitable.*
☒	☐	For Bayesian analysis, information on the choice of priors and Markov chain Monte Carlo settings
☒	☐	For hierarchical and complex designs, identification of the appropriate level for tests and full reporting of outcomes
☒	☐	Estimates of effect sizes (e.g. Cohen's d, Pearson's r), indicating how they were calculated

Our web collection on statistics for biologists contains articles on many of the points above.

Software and code

Policy information about availability of computer code

Data collection	No novel custom scripts were used. 100,000 Genomes data is processed according to a generic pipeline described here https://www.genomicsengland.co.uk/?wpdmdl=15664 . Data was annotated with Variant Effect Predictor release 98 (which includes for SPLICE AI and CADD annotation), Manta 1249, Canvas 1.3.1, Exomiser 12.1.0, and Mutect2 4.1.0.0 was applied using GATK 4.1.4.1
Data analysis	GraphPad Prism 8 software (GraphPad Software Inc., CA) and ImageJ v.2.0.0 software (NIH, USA)

For manuscripts utilizing custom algorithms or software that are central to the research but not yet described in published literature, software must be made available to editors and reviewers. We strongly encourage code deposition in a community repository (e.g. GitHub). See the Nature Portfolio guidelines for submitting code & software for further information.

Data

Policy information about availability of data

All manuscripts must include a data availability statement. This statement should provide the following information, where applicable:

- Accession codes, unique identifiers, or web links for publicly available datasets
- A description of any restrictions on data availability
- For clinical datasets or third party data, please ensure that the statement adheres to our policy

The datasets analysed during the current study (primary data from the 100,000 Genomes Project) are held in a secure Research Environment and are available to registered users. https://www.genomicsengland.co.uk/about-gecip/for-gecip-members/data-and-data-access

March 2021

Field-specific reporting

Please select the one below that is the best fit for your research. If you are not sure, read the appropriate sections before making your selection.

- [x] Life sciences
- [ ] Behavioural & social sciences
- [ ] Ecological, evolutionary & environmental sciences

For a reference copy of the document with all sections, see nature.com/documents/nr-reporting-summary-flat.pdf

Life sciences study design

All studies must disclose on these points even when the disclosure is negative.

Sample size	This is a descriptive study illustrating a real world example of WGS in a rare disease clinic. We included all patients attending our clinical service who consented to be included in the 100,000 Genomes study. No statistical analysis was used to determine this sample size.
Data exclusions	None
Replication	This is a unique cohort and it was not possible o obtain this level of clinical detail on another group of patients with suspected mitochondrial disease who had undergone WGS
Randomization	This is not relevant as the basis of the study is that all patients underwent the same diagnostic process. All the experiments reported in this paper were to verify the effect of variants identified in specific patients. For each experiment we had only one clinical case and a heathy control group, including 2 or 5 biologically independent controls. The controls were chosen to match the sex and age of the patient sample.
Blinding	Blinding is not possible as the hypothesis is based on detailed interrogation of the patients specific presentation. i.e., knowledge of each specific patient, including physical examination and review of investigations was a necessary part of the methodology for the clinician involved in reviewing data. The bioinformatician was blinded to the patients identities. Interpretation of histopathology was performed by a senior pathologist blinded to patient identity and clinical data.

Reporting for specific materials, systems and methods

We require information from authors about some types of materials, experimental systems and methods used in many studies. Here, indicate whether each material, system or method listed is relevant to your study. If you are not sure if a list item applies to your research, read the appropriate section before selecting a response.

Materials & experimental systems

n/a	Involved in the study	
[ ]	[x]	Antibodies
[ ]	[x]	Eukaryotic cell lines
[x]	[ ]	Palaeontology and archaeology
[x]	[ ]	Animals and other organisms
[ ]	[x]	Human research participants
[ ]	[x]	Clinical data
[x]	[ ]	Dual use research of concern

Methods

n/a	Involved in the study	
[x]	[ ]	ChIP-seq
[x]	[ ]	Flow cytometry
[x]	[ ]	MRI-based neuroimaging

Antibodies

Antibodies used	Primary antibodies used: COX7B (ab137094, Abcam); ATP5A, UQCRC2, COXII, SDHB and NDUFB8 (Total OXPHOS Human WB Antibody Cocktail- ab110411, Abcam); SDHA (14865-1-AP Proteintech); ß-actin (4970, Cell Signalling) and GAPDH (AM4300, ThermoFisher Scientific); MHCD (NCL-MHCd, Leica Biosystems) and MHCN (NCL-MHCn, Leica Biosystems)<br>Secondary antibodies used: IRDye® 800CW Goat anti-Mouse IgG (926-32210, Li-cor Biosciences); IRDye® 680LT Goat anti-Mouse IgG (926-68020, Li-cor Biosciences); IRDye® 800CW Goat anti-Rabbit IgG (926-32211, Li-cor Biosciences) and IRDye® 680RD Goat anti-Rabbit IgG Secondary Antibody (926-68071, Li-cor Biosciences)
Validation	COX7B (ab137094, Abcam) - According to manufacturer's website, the antibody was validated in COX7B knockout in HeLa cells<br><br>Total OXPHOS Human WB Antibody Cocktail (ab110411, Abcam) - According to manufacturer's protocol, the cocktail is suitable for Western Blotting analysis of the relative levels of the 5 OXPHOS complexes in mitochondrial preparations from human sources. The company does not provide a validation western blot for this antibody. However, on the company website it has been referenced in 348 publications.<br><br>SDHA (14865-1-AP Proteintech) - According to manufacturer's protocol, antibody has been validated on human samples. Company website reports three publications where the antibody was validated by protein knockdown in human samples.<br><br>ß-actin (4970, Cell Signalling) - Data on HeLa and A431 cell lines are reported in the manufacturer's website. According to

manufacturer's website, the use of this antibody for western blot analysis on human samples has reported in 221 publications.

GAPDH (AM4300, ThermoFisher Scientific) - According to manufacturer's website, the use of this antibody for western blot analysis has reported in 460 publications.

The antibodies against fast, slow, developmental (embryonic) and neonatal (fetal) myosin heavy chains (NCL-MHCf, NCL-MHCs, NCL-MHCd and NCL-MHCn) were validate in the diagnostic muscle pathology laboratory (Dubowitz Neuromuscular Centre). The antibodies against Fast 2A (7.5.2B, Draeger 1987) was obtained as a gift from Robin Fitzsimons and 2X (6H1, Lucas 2000) was obtained from DSHB, both of which were initially optimised in the diagnostic laboratory using a cohort of minimal change and dystrophic muscle biopsies.

Eukaryotic cell lines

Policy information about cell lines

Cell line source(s)	All cells used in this study were primary patient-derived.
Authentication	All experiments were performed on primary patient-derived fibroblasts. Cells used were not authenticated.
Mycoplasma contamination	All cells were tested negative for mycoplasma contamination
Commonly misidentified lines (See ICLAC register)	*Name any commonly misidentified cell lines used in the study and provide a rationale for their use.*

Human research participants

Policy information about studies involving human research participants

Population characteristics	We report a cohort of 102 participants of whom 56% [57/102] female, 44% [45/102] male). The age range was 17y-81y with a mean age of 47.3. All patients had a working diagnosis of 'suspected mitochondrial disease.' This was not a clinical trial/ interventional study and we did not gather information regarding treatment history.
Recruitment	Potential participants were identified during a weekly clinical team meeting and recruited to the 100,000 Genomes Study during clinic visits. Potential self-selection bias is relevant as patients had to attend clinic to be recruited, therefore more disabled or less motivated patients may have been selected against. We ameliorated this by supplying hospital transport and consenting patients during inpatient admissions. In our experience most very disabled patients were consented for by legal guardians.
Ethics oversight	Cambridge REC committee oversaw 100,000 Genomes project. (East of England – Cambridge South (REC Ref 14/EE/1112)). The present sub-study was undertaken as part of the MRC International Centre for Genomic Medicine in Neuromuscular Disease (ICGNDM) which was approved by the relevant REC (London - Camberwell St Giles Research Ethics Committee (REC Ref 19/LO/1796)). Informed consent was obtained for all patients in this sub-study, as well as consent to publish potentially identifying information.

Note that full information on the approval of the study protocol must also be provided in the manuscript.

Clinical data

Policy information about clinical studies

All manuscripts should comply with the ICMJEguidelines for publication of clinical research and a completedCONSORT checklist must be included with all submissions.

Clinical trial registration	NA
Study protocol	NA
Data collection	Data was analyzed between Jan 2020 and Nov 2021
Outcomes	NA
